# Transcriptome analysis reveals a major impact of JAK protein tyrosine kinase 2 (Tyk2) on the expression of interferon-responsive and metabolic genes

**DOI:** 10.1186/1471-2164-11-199

**Published:** 2010-03-25

**Authors:** Claus Vogl, Thomas Flatt, Bernd Fuhrmann, Elisabeth Hofmann, Barbara Wallner, Rita Stiefvater, Pavel Kovarik, Birgit Strobl, Mathias Müller

**Affiliations:** 1 Institute of Animal Breeding and Genetics, Department of Biomedical Sciences, University of Veterinary Medicine Vienna, Veterinärplatz 1, A-1210 Vienna, Austria; 2 Institute of Population Genetics, Department of Biomedical Sciences, University of Veterinary Medicine Vienna, Veterinärplatz 1, A-1210 Vienna, Austria; 3 Department of Microbiology and Immunology, Max F Perutz Laboratories, University of Vienna, Dr Bohr-Gasse 9, A-1030 Vienna, Austria; 4 Biomodels Austria, University of Veterinary Medicine Vienna, Veterinärplatz 1, A-1210 Vienna, Austria

## Abstract

**Background:**

Tyrosine kinase 2 (Tyk2), a central component of Janus kinase/signal transducer and activator of transcription (JAK/STAT) signaling, has major effects on innate immunity and inflammation. Mice lacking Tyk2 are resistant to endotoxin shock induced by lipopolysaccharide (LPS), and Tyk2 deficient macrophages fail to efficiently induce interferon α/β after LPS treatment. However, how Tyk2 globally regulates transcription of downstream target genes remains unknown. Here we examine the regulatory role of Tyk2 in basal and inflammatory transcription by comparing gene expression profiles of peritoneal macrophages from Tyk2 mutant and wildtype control mice that were either kept untreated or exposed to LPS for six hours.

**Results:**

Untreated Tyk2-deficient macrophages exhibited reduced expression of immune response genes relative to wildtype, in particular those that contain interferon response elements (IRF/ISRE), whereas metabolic genes showed higher expression. Upon LPS challenge, IFN-inducible genes (including those with an IRF/ISRE transcription factor binding-site) were strongly upregulated in both Tyk2 mutant and wildtype cells and reached similar expression levels. In contrast, metabolic gene expression was strongly decreased in wildtype cells upon LPS treatment, while in Tyk2 mutant cells the expression of these genes remained relatively unchanged, which exaggerated differences already present at the basal level. We also identified several 5'UR transcription factor binding-sites and 3'UTR regulatory elements that were differentially induced between Tyk2 deficient and wildtype macrophages and that have not previously been implicated in immunity.

**Conclusions:**

Although Tyk2 is essential for the full LPS response, its function is mainly required for baseline expression but not LPS-induced upregulation of IFN-inducible genes. Moreover, Tyk2 function is critical for the downregulation of metabolic genes upon immune challenge, in particular genes involved in lipid metabolism. Together, our findings suggest an important regulatory role for Tyk2 in modulating the relationship between immunity and metabolism.

## Background

The first crucial step in successfully fighting infections is the sensing of microbial products by innate immune cells, e.g. macrophages and dendritic cells [1]. Toll-like receptors (TLRs) sense microbial products and initiate a cascade of signaling events to alert the immune defense. Members of the TLR family recognize a range of pathogen-associated molecular patterns produced by bacteria, protozoa, fungi, or viruses [2,3]. One of the best studied examples for a pathogen associated pattern is bacterial lipopolysaccharide (LPS). Binding of LPS to the TLR4 complex of the host activates two signaling cascades via different adaptor proteins leading to the activation of two main transcription factors: NFκB, a key transcription factor regulating pro-inflammatory genes, and interferon (IFN) regulatory factor 3 (IRF3), which induces IRF3-responsive genes, most prominently IFNβ. IFNβ and other cytokines subsequently act in an autocrine/paracrine manner to induce the expression of immune responsive genes [4,5].

A major component of IFNα/β signaling is Tyrosine kinase 2 (Tyk2), a member of the Janus kinase (JAK) family [6]. Activated JAKs phosphorylate members of the signal transducer and activator of transcription (STAT) family, which mediate signals from a large number of cytokines and growth factors [7,8]. Activation of JAK1 and Tyk2 by IFNα/β stimulation leads to a series of phosphorylation events, causing the formation of IFN stimulated gene factor 3 (ISGF3) complexes that consist of STAT1, STAT2 and IFN regulatory factor 9 (IRF9). Subsequently, ISGF3 translocates to the nucleus and binds to a specific transcription factor binding-site (TFBS), the IFN stimulated response element (IRF/ISRE), which in turn activates transcription of several hundred IFN-responsive genes. To a lesser extent, these signaling events also lead to the formation of STAT1 and STAT3 homo- and heterodimers that bind another TFBS, the IFNγ activated sequence (GAS TFBS) [9].

In mice, Tyk2 is only partially required for IFNα/β signaling, where it mainly serves to amplify the immune response [10,11]. We and others have previously shown a surprisingly low susceptibility of Tyk2 knockout mice to LPS-induced endotoxin shock, despite normal production of proinflammatory cytokines (the interleukins IL6, IL1B) and tumor necrosis factor α (TNFα) [12,13]. *In vitro*, Tyk2 mutant peritoneal macrophages fail to efficiently induce IFNα/β and nitric oxide (NO) production upon LPS treatment. In addition, Tyk2 deficiency influences the basal level of several IFN-responsive genes [10,12]. However, despite the importance of Tyk2 in mediating immune and inflammatory responses, how this JAK regulates global transcription of downstream target genes remains largely unknown.

Here we compare the genome-wide expression profiles of Tyk2 mutant and wildtype mouse peritoneal macrophages with and without activation by LPS using microarrays. In particular, we focus on IFN-responsive genes and their putative regulators and use bioinformatic analysis to examine how the observed changes in gene expression relate to gene ontology categories and *cis*-regulatory elements (i.e., 5'UR TFBSs and 3'UTR elements) [14]. We find that Tyk2 is essential for mediating the full LPS response and for baseline expression of IFN-inducible genes, but not for the LPS-induced upregulation of IFN-responsive genes. Moreover, we show that LPS challenge suppresses the expression of genes involved in metabolism and we establish a critical role for Tyk2 function in this downregulation.

## Results

### Effects of Tyk2 and LPS on gene expression

To determine the effects of Tyk2 and LPS on gene expression we used CodeLink Mouse Whole Genome Arrays on mRNA isolated from Wt (C57BL/6) and Tyk2-/- (on a C57BL/6 background) macrophages, either stimulated with LPS for six hours or kept untreated. After filtering and quality control, we retained 7546 genes for statistical and bioinformatic analysis (see Methods).

**Figure 1:**
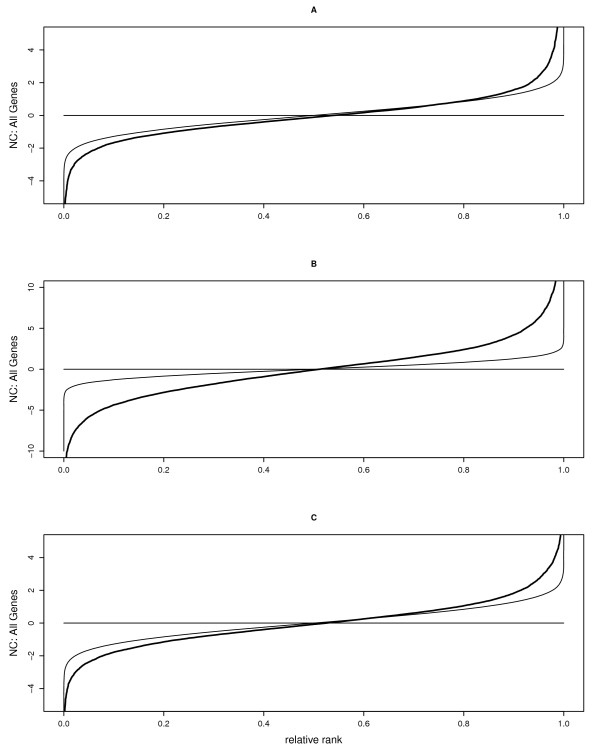
**Effects of Tyk2 and LPS on overall gene expression**. Effects of (A) Tyk2 genotype (Wt minus Tyk2-/-), (B) LPS treatment (6 hours of LPS minus control), and (C) genotype by treatment interaction (difference in LPS induction between Wt and Tyk2-/-) on expression levels of all genes, plotted as normed effect coefficients (NCs) (y-axis) against relative ranks (absolute rank divided by the number of genes; x-axis). Thick solid lines represent NCs; thin solid lines the values expected under the assumption of the null hypothesis and normality.

**Figure 2:**
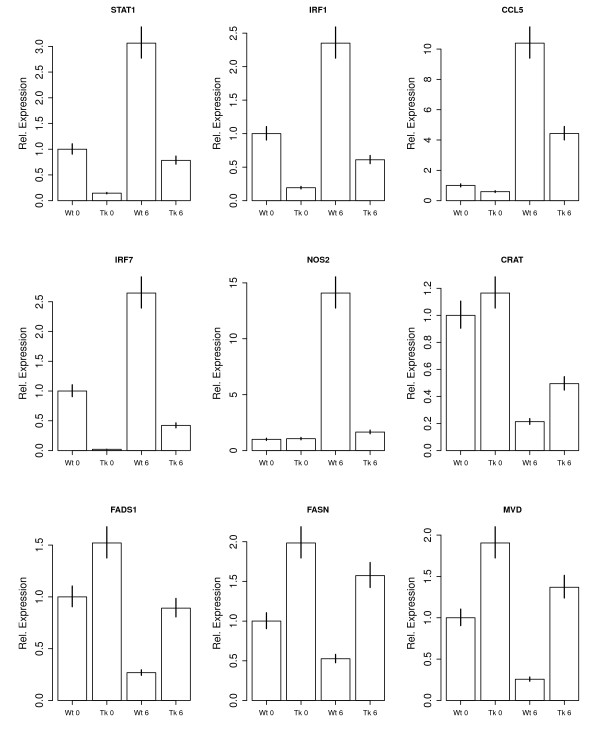
**Effects of Tyk2 and LPS on relative expression levels of immune genes and lipid genes**. Representative examples of the effects of genotype (Wt vs.Tyk2-/- (Tk)) at the basal level (0) or after six hours of LPS treatment (6). Five genes (STAT1, IRF1, CCL5, IRF7, NOS2) are immunity annotated and IFN-responsive genes; four genes (CRAT, FADS1, FASN, MVD) are annotated for lipid metabolism. Expression levels, as determined by microarray fluorescence intensities, are shown relative to the mean basal level in the Wt. Error bars represent standard errors. All genes were validated with RT-qPCR (see Materials and Methods).

At the basal level, in the absence of LPS stimulation, we found that 428 (6%) of 7546 genes analyzed were differentially expressed between Wt and Tyk2-/- macrophages (effect of genotype, Figure 1A; full dataset available at Gene Expression Omnibus http://www.ncbi.nlm.nih.gov/geo/, GEO accession number GSE19733). Among the genes with the largest expression differences between genotypes, many are known to be involved in immune function. The majority of these genes was downregulated in Tyk2-/- relative to Wt cells, including for example IFNγ- inducible protein 47 (IFI47), IFN regulatory factor 7 (IRF7), and 2'-5' oligoadenylate synthetase-like genes 1 and 2 (OASL1, OASL2), as well as three out of five (STAT1, IRF1, and IRF7) IFN-responsive genes shown in Figure 2.

**Figure 3:**
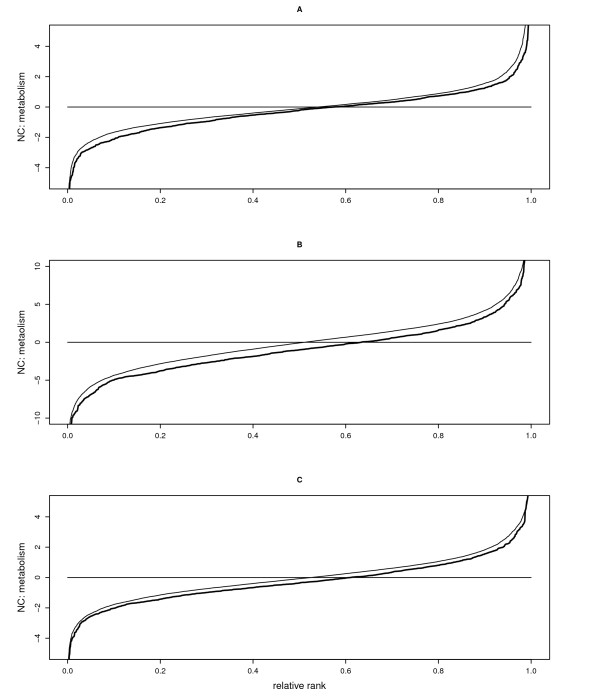
**Effects of Tyk2 and LPS on metabolic gene expression**. Effects of (A) Tyk2 genotype (Wt minus Tyk2-/-), (B) LPS treatment (6 hours of LPS minus control), and (C) genotype by treatment interaction (difference in LPS induction between Wt and Tyk2-/-) on expression levels of genes annotated for metabolism, plotted as NCs (y-axis) against relative ranks (absolute rank divided by the number of genes; x-axis). Thick solid lines represent NCs; thin solid lines the values of all genes (see Figure 1) in order to highlight effects on metabolic genes relative to all genes analyzed.

Consistent with this, gene ontology (GO) analysis showed that gene classes involved in immunity were significantly downregulated in Tyk2-/- relative to Wt cells (Table 1A). Interestingly, genes belonging to metabolic gene classes showed higher expression in Wt relative to Tyk2-/-, including genes in the classes "metabolism", "lipid biosynthesis", "sterol biosynthesis", and "cholesterol biosynthesis" (Table 1A). Overall, we observed a small but significant shift in gene expression towards lower values in Tyk2-/- cells, suggesting the involvement of the majority of metabolic genes in this shift (Figure 3A). This relatively small but consistent effect can also be seen at the level of individual genes, for example for the lipid genes shown in Figure 2. Thus, in macrophages, Tyk2 is not only required for basal expression of immune genes but also for the maintenance of metabolic genes at a low level of expression in an uninduced state.

**Table 1:** Results of gene ontology analysis using the "biological process" annotation

GO category	# genes	ER	NC	p-value
**(A)**				

humoral defense mechanism sensu Vertebrata	11	1.58	4.3	0

antigen presentation	11	1.54	4.02	0

defense response	46	1.34	2.72	0

immune response	102	1.28	2.31	0

positive regulation of transcription				

DNA-dependent	13	1.19	1.65	0.0013

regulation of apoptosis	45	1.11	0.94	0.0005

protein ubiquitination	78	1.06	0.58	0.0049

protein modification	71	1.06	0.57	0

transcription	449	1.04	0.34	0

regulation of transcription DNA.dependent	586	1.03	0.29	0

transport	576	0.97	-0.29	0.0002

electron transport	138	0.95	-0.49	0.002

metabolism	151	0.92	-0.8	0

sodium ion transport	22	0.88	-1.2	0.0025

biosynthesis	24	0.87	-1.26	0.0009

fatty acid biosynthesis	15	0.85	-1.57	0.0011

lipid biosynthesis	34	0.83	-1.77	0

steroid biosynthesis	20	0.83	-1.79	0

cholesterol biosynthesis	12	0.79	-2.21	0

sterol biosynthesis	13	0.77	-2.5	0

**(B)**				

inflammatory response	46	2.24	7.55	0

chemotaxis	30	2	6.48	0

immune response	102	1.93	6.18	0

sensory perception	42	1.64	4.66	0

defense response to bacteria	11	1.63	4.61	0.0001

defense response	46	1.37	2.97	0

cell surface receptor linked signal transduction	32	1.28	2.28	0.001

sensory perception of smell	70	1.25	2.08	0

cell proliferation	42	1.24	2.03	0.0008

signal transduction	222	1.21	1.79	0

regulation of apoptosis	45	1.2	1.73	0

G-rotein coupled receptor protein signaling pathway	166	1.15	1.32	0

regulation of transcription	119	1.1	0.87	0

regulation of transcription DNA.dependent	586	1.04	0.38	0

electron transport	138	0.87	-1.26	0

metabolism	151	0.82	-1.91	0

lipid metabolism	59	0.79	-2.21	0

lipid biosynthesis	34	0.77	-2.49	0

fatty acid metabolism	32	0.71	-3.15	0

**(C)**				

negative regulation of progression through cell cycle	26	1.17	0.95	0.0022

anti apoptosis	30	1.17	0.92	0.0015

metabolism	151	0.92	-0.47	0.0009

protein biosynthesis	180	0.92	-0.52	0

cell proliferation	42	0.87	-0.8	0.0021

fatty acid metabolism	32	0.85	-0.95	0.0015

rRNA processing	29	0.85	-0.97	0.0019

lipid biosynthesis	34	0.84	-1.02	0.0004

steroid biosynthesis	20	0.83	-1.09	0.0037

sterol biosynthesis	13	0.8	-1.34	0.0039

cholesterol biosynthesis	12	0.79	-1.43	0.003

Within the "biological process" annotation, gene classes (412 in total) were separately contrasted with all other classes using *t-*tests on standardized coefficients. (A) Basal differences between genotypes, (B) LPS induction in Wt, and (C) differences between genotypes in LPS induction (genotype by treatment interaction). ER, approximate mean expression ratio, NC, mean normed coefficients.

In contrast to the relatively small number of differences in baseline expression between Wt and mutant cells, LPS treatment changed the transcriptional status of Wt macrophages dramatically: 3622 genes (48%) were either up- or downregulated upon exposure to LPS, including many genes involved in immunity (effect of LPS treatment, Figure 1B; GEO accession number GSE19733). This massive change in transcriptional status is also reflected in the large distance between the observed curve and the curve showing the theoretical expectation in Figure 1B. Among the genes upregulated by LPS, several are known to also be induced by cytokines, chemokines, or cellular stressors, including the interleukins IL1A, IL1B, IL12A, the IFN stimulated gene ISG20, and TNFα, as well as all five IFN-responsive genes shown in Figure 2. In contrast, genes involved in lipid metabolism were typically downregulated in Wt cells (see Figures 2 and 3B). Similar to the situation at the basal level, this downward shift in expression involved almost all genes (Figure 3B).

In total, LPS treatment influenced 19 GO classes, with strong upregulation of genes involved in inflammatory responses and chemotaxis and downregulation of genes involved in metabolism, indicating a negative relationship between immune response and metabolism (Table 1B; see Figure 3B for metabolic genes). In Tyk2 mutant cells, however, only 2297 genes (30%) were influenced by LPS, suggesting that Tyk2-/- cells respond overall much less to LPS than Wt cells (GEO accession number GSE19733).

The observation that Tyk2-/- macrophages might exhibit an impaired LPS response was confirmed when we analyzed differences in how genotypes react to LPS challenge: 1202 genes (16%) showed a difference in LPS induction between Wt and Tyk2 mutant (effect of genotype × treatment interaction, Figure 1C, GEO accession number GSE19733). For example, among the differentially induced immune genes, LPS caused a much weaker induction of the interferon-stimulated protein ISG20 and the NO synthase NOS2 in Tyk2-/- than in Wt cells, but a stronger induction of IFI47 and IRF7 relative to Wt (Figure 2).

Overall, we found that eleven classes of genes were differentially induced by LPS between genotypes, including weaker upregulation of genes involved in apoptosis and negative cell cycle control and weaker suppression of metabolic gene expression in Tyk2-/- relative to Wt cells (Table 1C; see Figures 2 and 3C for metabolic genes). Again, we found that this downward shift in gene expression involved the majority of all genes (Figure 3C). Most notably, genes involved in "cholesterol biosynthesis", "sterol biosynthesis", "steroid biosynthesis", and "lipid biosynthesis" were much less strongly suppressed upon LPS challenge in Tyk2-/- than in Wt macrophages. Metabolic genes were therefore downregulated by LPS in Wt cells, but their expression levels remained closer to the basal, uninduced state in Tyk2-/- macrophages. RT-qPCR broadly confirmed the effects of Tyk2 on immune and metabolic genes (Additional File 1). Thus, Tyk2 is required for LPS-induced upregulation of immune and other genes and for the suppression of metabolic genes upon LPS challenge (also see Additional File 1).

### Transcription factor binding-sites

The expression of the about 25,000 genes in mammalian genomes is regulated by many fewer transcription and other regulatory factors. We therefore related our microarray expression data to information on TFBSs upstream of the transcription start site [14]. Since TFBSs are often short and relatively uninformative, and because many putative TFBSs are biologically irrelevant, we extracted sequences from the 5' upstream region (5'UR) that are conserved between human and mouse, as such phylogenetic conservation might indicate functional importance in transcriptional control. This bioinformatics analysis identified 174 classes of putative 5'UR TFBSs.

**Figure 4:**
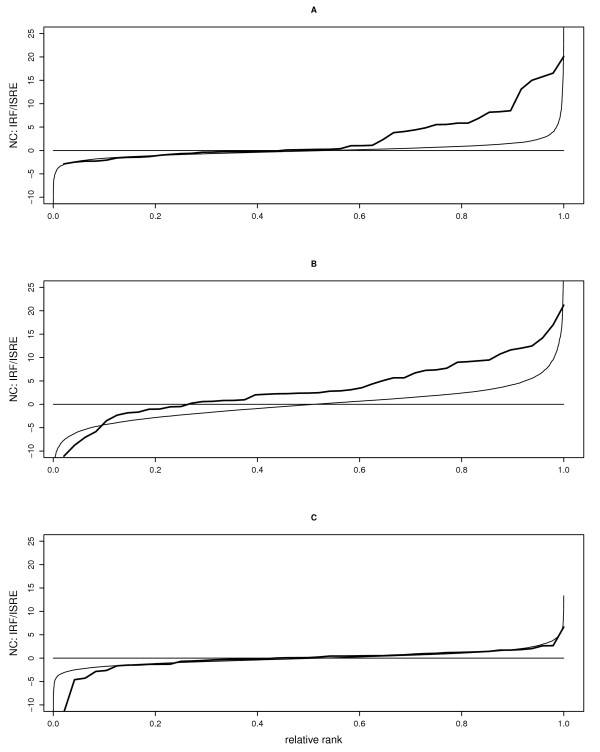
**Effects of Tyk2 and LPS on genes with IRF/ISRE TFBS**. Effects of (A) Tyk2 genotype (Wt minus Tyk2-/-), (B) LPS treatment (6 hours of LPS minus control), and (C) genotype by treatment interaction (difference in LPS induction between Wt and Tyk2-/-) on expression levels of genes containing the IRF/ISRE TFBS, plotted as NCs (y-axis) against relative ranks (absolute rank divided by the number of genes; x-axis). Thick solid lines represent NCs; thin solid lines the values of all genes (see Figure 1) in order to highlight the effects on genes with IRF/ISRE TFBS relative to all genes analyzed.

At the basal level, only genes that contain the IRF/ISRE TFBS were differentially regulated between genotypes; genes with GAS elements showed no significant difference between genotypes (Figure 4A, Table 2A, Additional File 2). Since the IRF/ISRE element is responsive to the ISGF3 complex, genes containing such an element are likely candidate targets of IFNα/β signaling. Tyk2-/- macrophages had on average an approximately 1.4 times reduced expression of genes with an IRF/ISRE TFBS as compared to Wt cells (Figure 4A, Table 2A, Additional File 2). Unlike the situation for metabolic genes, this difference between genotypes was not caused by the majority of genes, but by a few genes with much higher expression in Wt than in Tyk2-/- (Figure 4A). Moreover, the expression of several immune genes, including Z-DNA binding protein 1 (ZBP1), guanylate binding protein 3 (GBP3), IFN-induced transmembrane protein 3 (IFITM3), and the interleukin 15 receptor α (IL15RA), was about three times higher in Wt than in Tyk2-/- cells (Additional File 1, GEO accession number GSE19733).

**Table 2:** Analysis of putative 5'UR TFBSs

TFBS	# genes	ER	NC	p-value
**(A)**				

IRF/ISRE	40	1.41	3.20	0.0011

**(B)**				

IRF/ISRE	40	1.53	3.99	0.0001

127	35	1.19	1.60	0.0006

CHX10	160	1.09	0.80	0.0001

IPF1 = PDX1	108	1.08	0.72	0.0010

TATA	280	1.07	0.67	0.0005

174	176	1.06	0.56	0.0009

NF-AT	519	1.03	0.25	0.0011

**(C)**				

115	33	1.13	0.73	0.0006

POU1F1	49	1.12	0.65	0.0019

IPF1 = PDX1	108	1.08	0.45	0.0003

FOXF2	171	1.07	0.40	0.0018

CHX10	160	1.06	0.35	0.0010

TATA	280	1.06	0.33	0.0005

We assigned genes to 174 classes of putative TFBSs, either identified by their name, the major transcription factor binding to them, or their number in Xie et al. [14]. The table shows the number of genes in each TFBS class, approximate mean expression ratios (ER), mean normed coefficients (NC), and p-values from *t*-tests. (A) Basal differences between genotypes, (B) LPS induction in Wt, and (C) differences between genotypes in LPS induction (genotype by treatment interaction).

LPS treatment influenced seven classes of genes in Wt macrophages (Figure 4B, Table 2B, Additional File 2), with two of them containing putative binding-sites for unknown transcription factors. Similar to the baseline differences in expression between genotypes, genes with the IRF/ISRE TFBS (e.g., gram negative binding protein 3 (GNBP3), cluster of differentiation 274 (CD274), and IL15RA) were strongly upregulated after six hours of LPS treatment. These genes were followed by an uncharacterized class of genes, either with CHX10 TFBS (e.g., β-site APP cleaving enzyme 1 (BACE1), the 14-3-3 gene Stratifin, the Myc associated factor X (MAX), and CD38) or containing homeodomain transcription factor IPF1/PDX1 motifs (e.g., Stratifin, the tripartite motif gene TRIM21, and the zinc finger transcription factor Zfp281). To our knowledge, neither CHX10 TFBS nor IPF1/PDX1 motifs have previously been implicated in the LPS response. Genes containing a TATA box were also significantly upregulated upon LPS exposure (especially the LIM homeodomain factor LHX4 and TNFα). Furthermore, we found evidence for weak upregulation of genes with conserved NF-AT motifs (Table 2B), with AP-1/Jun and c-REL consensus sequences (using a significance level of α = 0.01 - see Methods for definition of cutoff criteria; Additional File 2), and with NFκB motifs (using α = 0.05; Additional File 2). Interestingly, we failed to observe LPS-induced downregulation of any gene class (either using FDR = 0.05 or α = 0.01 as cutoffs; Table 2B, Additional File 2).

Six classes of genes differed in their induction by LPS between genotypes, and for all these classes induction by LPS was lower in Tyk2-/- than in Wt cells (Table 2C, Additional File 2). Genes containing the pituitary-specific transcription factor POU1F1 motif showed the most pronounced differences in mean expression, whereas genes with a IPF1/PDX1 motif (especially Stratifin) or a TATA box (especially LHX4 and NOS2) showed the highest statistical significance (Table 2C, Additional File 2). However, despite these genotypic differences in inducibility, LPS induced on average similar expression of genes with an IRF/ISRE TFBS in Wt and Tyk2-/- macrophages (Figure 4C, Table 2C, Additional File 2). Upon LPS challenge, Wt and Tyk2-/- cells therefore reached qualitatively and quantitatively similar expression levels of genes that contain the IRF/ISRE TFBS. These results suggest that Tyk2 is functionally required for the baseline expression of genes with an IRF/ISRE TFBS, but dispensable for their LPS-induced upregulation.

Since the above analysis of IRF/ISRE TFBSs was based on very stringent criteria and thus likely to miss many known IFN-inducible genes (see Methods), we also produced a list of 187 IFN-responsive genes from the literature and asked how many of those genes showed expression changes in our microarray experiment and contain IRF/ISRE or GAS TFBSs.

After filtering of lowly expressed genes, 137 genes remained in the analysis. At the basal level, we found that this class of IFN-inducible genes was on average downregulated relative to all other genes in Tyk2-/- versus Wt macrophages (Additional File 3). While the mean expression ratios relative to Wt were quite small, the average difference was caused by a few genes of large effect, with 23 genes being significantly differentially expressed (Additional File 3). The genes that showed the largest expression differentials were IRF7, IFN-inducible proteins 1 and 205 (IFI1, IFI205), and transporter associated with Ag processing 1 (TAP1), thereby confirming that Tyk2 is required for baseline expression of IFN-inducible genes. Upon LPS stimulation, IFN-inducible genes were on average much more strongly upregulated than other genes (74 genes in Wt; Additional File 3). Again, this pattern was mainly caused by a few genes with extreme effects, including IL1B, chemokine (C-C motif) ligands 2 and 7 (CCL2, CCL7), and chemokine (C-X-C motif) ligand 2 (CXCL2). As in our analysis of genes with the IRF/ISRE TFBS, the average induction of IFN-inducible genes by LPS was similar between Tyk2-/- and Wt macrophages, with both cell types reaching qualitatively identical expression levels (Additional File 3). To validate these microarray results we performed RT-qPCR on nine IFN-inducible genes and found a good agreement with respect to approximate fold induction (Additional File 1).

IFN-responsive genes contained significantly more IRF/ISRE and GAS TFBSs than other genes, although enrichment was only modest for the latter. The criteria of inclusion, i.e. the length of the 5'UR and the counting of only TFBSs conserved between human and mouse influenced the results considerably. As expected, conservation of TFBSs among species decreased the number of false positives, whereas a long 5'UR stretch or no conservation increased it (Additional File 3).

### 3' UTR regulatory elements

As mRNA levels are not only known to be regulated by 5'UR TFBSs, but also post-transcriptionally by regulatory elements in the 3'untranslated region (UTR), we were interested in relating the observed expression differences to putative 3'UTR regulatory sequences. We focused on two not mutually exclusive classes of regulatory sequences: (i) conserved 3'UTR motifs and (ii) 3'UTR microRNA (miRNA) cognate sequences.

To examine motifs in the 3'UTR we analyzed our data with respect to 175 classes of conserved putative 3'UTR regulatory sequences defined by Xie et al. [14] (Table 3, Additional File 4). As compared to 5'UR TFBSs, we found many more gene classes to be differentially expressed (Table 3, Additional File 4). This might simply be due to a larger number of genes containing specific 3'UTR motifs than 5'UR TFBSs. Alternatively, 3'UTR elements might be more conserved, or easier to detect, than 5'UR TFBSs. We observed four classes of motifs that were differentially regulated between genotypes at the basal level (Table 3A), 37 that were up- or downregulated after LPS treatment in Wt (Table 3B), and three that showed differences in LPS inducibility between genotypes (Table 3C). Genes with AU-rich elements (m2/ARE) were the most significantly upregulated upon LPS treatment (Table 3B, Additional File 5). Similar to what we observed for IFN-responsive genes or those with IRF/ISRE TFBSs, this pattern was mainly caused by a few genes with extreme effects on expression, such as IL1A, IL1B, CXCL2, and TNFα, which were among the most responsive genes (GEO accession number GSE19733). In contrast, LPS caused downregulation of a large class of genes containing poly-A signal (m1/poly-A) motifs (Table 3B). However, genes with m2/ARE or m1/poly-A motifs neither differed in baseline expression, nor in LPS inducibility between Wt and Tyk2-/- macrophages.

**Table 3:** Analysis of 3'UTR regulatory sequences

3'UTR motif	gene #	ER	NC	p-value
**(A)**				

m57	110	1.04	0.39	0.0037

m11	623	0.98	-0.23	0.0026

m43	254	0.96	-0.33	0.0009

o7	207	0.96	-0.33	0.0058

**(B)**				

m53	42	1.30	2.60	0.0076

o34	75	1.13	1.22	0.0006

m35	54	1.13	1.18	0.0026

m103	41	1.11	1.05	0.0048

m33	119	1.11	1.04	0.0026

m95	56	1.11	1.03	0.0004

m16	213	1.10	0.99	0.0000

o25	105	1.09	0.86	0.0011

m85	82	1.09	0.84	0.0043

m2/ARE	675	1.09	0.82	0.0000

m67	164	1.07	0.68	0.0004

o18	278	1.07	0.65	0.0001

m50	108	1.07	0.64	0.0081

m100	70	1.06	0.60	0.0079

o43	142	1.05	0.49	0.0021

m30	85	1.05	0.49	0.0133

m70	136	1.05	0.48	0.0108

o12	196	1.05	0.48	0.0012

m24	332	1.05	0.47	0.0001

o15	253	1.04	0.43	0.0011

m3	481	1.04	0.43	0.0000

m20	262	1.04	0.40	0.0024

o38	142	1.04	0.38	0.0063

m29	232	1.04	0.37	0.0013

m41	224	1.04	0.36	0.0025

o32	155	1.04	0.36	0.0067

m13	1015	1.03	0.30	0.0000

m4	625	1.03	0.29	0.0000

o21	363	1.03	0.26	0.0011

m19	308	1.02	0.25	0.0028

m9	942	1.02	0.24	0.0000

m31	347	1.02	0.21	0.0018

m25	488	1.02	0.21	0.0006

m8	337	1.02	0.17	0.0077

m26	383	1.02	0.15	0.0017

m5	1175	1.01	0.06	0.0000

m1/poly-A	1938	0.95	-0.54	0.0091

**(C)**				

m91	66	1.1	0.56	0.0046

m13	1015	1.01	0.06	0.0016

m9	942	1.01	0.06	0.0016

A total of 175 3'UTR motis were identified as described in Xie et al. [14]. The table shows the number of genes in each class of motif, approximate mean expression ratios (ER), mean normed coefficients (NC), and p-values from *t*-tests. (A) Basal differences between genotypes, (B) LPS induction in Wt, and (C) differences between genotypes in LPS induction (genotype by treatment interaction).

A special class of 3'UTR regulatory elements important in the regulation of gene expression are the targets of miRNAs, short single-stranded RNA molecules consisting of 21-24 nucleotides. To analyze miRNA cognate motifs we related known miRNAs from miRBase http://www.mirbase.org/[15-17] to the octameric sequences in [14]. Using this approach, we were able to uniquely define different cognate 3'UTR sequences (which might otherwise have been lumped into a single class in the analysis above). Out of 111 such sequences, we found three miRNA cognate motif classes to be differentially expressed at the basal level, three classes to be influenced by LPS in Wt cells, and three classes to differ in their LPS inducibility between genotypes (Additional File 6). As far as we know, none of these miRNA motifs has been previously implicated in the IFN or LPS response. However, since conserved 3'UTR elements and miRNA consensus sites are still poorly characterized, it is difficult to functionally interpret these results. Nonetheless, our observations provide strong evidence for an involvement of such regulatory sequences in the LPS response of Wt and Tyk2-/- macrophages.

## Discussion

In this microarray study we have investigated the role of the JAK protein kinase family member Tyk2 in basal and LPS-induced gene expression in mouse peritoneal macrophages. While several previous studies have demonstrated that Tyk2 is an important regulator of immune and inflammatory responses [10,12,18], here we show how Tyk2 globally affects genome-wide transcript levels.

At the basal uninduced level, we found subtle but significant differences in gene expression between Wt and Tyk2-/- macrophages. Relative to Wt, Tyk2-deficient macrophages displayed reduced expression of many immune genes, in particular some genes that contain IFN response elements (IRF/ISRE) in their 5'UR, suggesting that Tyk2 deficiency compromises the type I IFN (i.e., IFNα/β) response. This observation confirms previous findings demonstrating that IFNα/β signaling is reduced in Tyk2-/- mice and that IFNα4 and IFNβ mRNA levels are diminished in Tyk2 null macrophages at the basal level [10-12].

In addition to the ISGF3 complex, type I IFNs also activate STAT1 homodimers, which induce gene expression via IFNγ activated sequence (GAS) elements. Furthermore, STAT1 homodimers are strongly activated by IFNγ, whose production and signaling is partially impaired in the absence of Tyk2 [10-12]. It is still controversial whether macrophages can produce significant amounts of biologically active IFNγ [19], however, cells might have been exposed to IFNγ during the generation procedure. We were therefore interested to determine if genes containing GAS TFBSs are also influenced by the absence of Tyk2. In contrast to the involvement of IRF/ISRE TFBS in the Tyk2 mediated response, we failed to find evidence for differential expression of genes containing GAS elements. Since the mouse genome contains very many such elements, not all of which are involved in gene regulation, it is possible that our bioinformatic analysis failed to find those GAS elements that are biologically relevant. Thus, while in our study reduced baseline expression of immunity related and IFN-responsive genes in Tyk2-deficient macrophages seems to be mainly mediated by IRF/ISRE TFBSs, the role of Tyk2 in GAS-driven gene regulation deserves further investigation.

We also found that Tyk2 is required for the full response of genes upon LPS challenge, as expected based on previous experiments [10,12]. LPS challenge affected the expression of about 48% of all genes in Wt cells, including many immune genes, but influenced only about 30% of all genes in Tyk2-/- cells. Consequently, Tyk2-deficient macrophages failed to induce many LPS-responsive transcripts, in particular immunity related genes. Surprisingly, however, IFN-responsive genes did not follow this trend: LPS challenge increased the expression of IFN-inducible genes to a similar extent in Wt and Tyk2-/- macrophages, suggesting that Tyk2 might not be critical for LPS-induced upregulation of IFN-responsive genes. Thus, although previous observations have found reduced levels of IFNα4 and IFNβ in Tyk2-/- macrophages [12], a high proportion of LPS-induced type I IFN signaling might be largely independent of Tyk2.

Several previous studies have examined IFN mediated immune and inflammatory transcription in bone marrow derived mouse macrophages (BMDMs) using microarrays [20,21]. Thomas et al. [20] studied Wt and IFNβ-/- cells at the basal level and after one and three hours of LPS treatment using Affymetrix microarrays. Many genes whose baseline levels were reduced by lack of IFNβ also had significantly lower levels in Tyk2-/- cells in our experiment. The overall correlation between expression differences for IFNβ-/- vs. Wt [20] and Tyk2-/- vs. Wt (our study) was *r *= 0.41 (see Additional File 7). Hence, the effect of IFNβ deficiency on baseline gene expression is similar to the lack of Tyk2. Thomas et al. [20] also identified many LPS-induced genes in Wt that we found to be induced in our study (overall correlation *r *= 0.48 - see Additional File 7). However, while IFNβ-/- macrophages showed an impaired LPS response for many IFN dependent genes [20], induction of these genes by LPS was generally unimpaired in Tyk2-/- macrophages in our experiment. A comparison of LPS inducibility between IFNβ-/- and Tyk2-/- macrophages only revealed a weak correlation of *r *= 0.03 (Additional File 7). Tyk2 therefore seems to be required for basal expression of IFNβ target genes but not for their induction by LPS. In another microarray study by Fleetwood et al. [21], genes whose basal expression levels depend on intact type I IFN signaling were characterized in BMDMs, either cultivated in the presence of macrophage colony stimulating factor (M-CSF, or CSF1) or granulocyte macrophage colony stimulating factor (GM-CSF). Most genes that were downregulated in IFNAR1-/- genotypes in both M-CSF and GM-CSF BMDMs were also decreased in Tyk2-/- relative to Wt cells in our experiment (Additional File 8), thus confirming our observation that Tyk2 is involved in basal IFNα/β signaling. Furthermore, the effects of basal type I IFN signaling on gene expression in the thioglycolate-elicited peritoneal macrophages used in our study were similar to those observed in the BMDMs used in [20] and [21].

Interestingly, our study also provides evidence for a previously unknown role of Tyk2 in regulating metabolism. Tyk2 mutant macrophages showed enhanced baseline expression of metabolic genes relative to Wt cells, including genes involved in steroid, sterol, and lipid metabolism. Upon LPS treatment, metabolic gene expression was strongly decreased in Wt macrophages, while in Tyk2-/- macrophages expression of these genes remained relatively unchanged. Thus, the differential LPS response of Wt versus mutant macrophages increased differences in gene expression already present at the basal level: the relatively low expression of metabolic genes in Wt cells was strongly suppressed by LPS, whereas in Tyk2-/- cells LPS suppressed the relatively high expression of metabolic genes much less. This effect pertained to most genes in the GO class "metabolism", and in particular to genes belonging to the classes "cholesterol biosynthesis", "sterol biosynthesis", "steroid biosynthesis", and "fatty acid metabolism" Taken together, these results suggest an involvement of Tyk2 in the regulation of general metabolism and of lipid metabolism in particular. Tyk2 might thus be an important mediator of the connection between immunity and metabolism.

Two not mutually exclusive mechanisms might account for the effects of Tyk2 on metabolic gene expression. On the one hand, Tyk2/IFN signaling might directly lead to decreased metabolism, possibly because upregulation of immune function shuts down other energetically demanding processes [22,23]. On the other hand, Tyk2 might exhibit pleiotropic effects on immunity and metabolism that are largely independent: a canonical IFN signaling function of Tyk2 might be associated with an independent role in metabolic regulation. Potentially consistent with either model, we observed that LPS treatment of macrophages reduces the expression of mitochondrial genes and that this downregulation is impaired in Tyk2-/- cells relative to Wt (GO annotation "cellular component", GO class "mitochondrion"; results not shown). Remarkably, dysfunctional mitochondrial respiration has been shown for Tyk2-deficient pro-B cells [24]. In a similar vein, Pitroda et al. [25] found that STAT1 knockdown is associated with alterations in the expression of genes involved in energy metabolism, including glycolysis, oxidative phosphorylation, and the citrate cycle. Tyk2/IFN signaling might thus regulate metabolism by targeting mitochondrial processes via STAT1.

Notably, we observed a particularly pervasive effect of Tyk2 on lipid and fatty acid metabolism in macrophages. This result fits well with previous studies that have reported intricate but not well understood interactions between immunity, inflammation, and lipid metabolism [for reviews see [21,22]]. For example, lipids can be sensed by and act on Toll-like receptors (TLRs) such as TLR4, probably because TLR agonists like LPS contain a biologically active lipid moiety [see [23]]. Moreover, macrophages and adipocytes are derived from a common ancestral progenitor and share several transcriptional features, with macrophages expressing some adipocyte specific genes and adipocytes expressing several macrophage specific genes, including IL6 and TNFα [see [23], and references therein]. However, little is currently understood about the downstream effects of impaired IFN signaling on lipid metabolism, as we have observed them in our experiments.

Although the biological details remain unclear, several studies have established a link between IFN signaling, innate immunity, and lipid metabolism. Mice deficient for interleukin 1 receptor antagonist α (IL1Ra), for instance, are lean, have impaired body fat accumulation, and exhibit reduced lipoprotein lipase activity [26], and TNFα can increase lipolysis and promote apoptosis of adipocytes [27]. Moreover, Zwaferink et al. [28] have recently reported that key lipogenic enzymes, including fatty acid synthase (FASN), are suppressed in IFNβ treated mouse BMDMs. Since we have observed a similar downregulation in Tyk2 mutant macrophages, and because Tyk2 deficiency is known to decrease IFNβ levels [12], it might be possible that Tyk2 influences lipid metabolism through a basal feed-forward loop between IFNβ and Tyk2.

Our findings are also consistent with our previous observation that Tyk2 modulates metabolic proteins in BMDMs before and after LPS treatment [29]. However, it remains possible that isolated mouse macrophages display somewhat abnormal metabolic behavior. We found most metabolic genes to be suppressed upon LPS treatment in Wt macrophages, but *in vivo *results suggest that Wt mice become hypermetabolic ninety minutes after LPS injection [30]. Thus, future studies will need to examine the detailed role of Tyk2 in regulating metabolism, both in macrophages and other cell types as well as *in vivo*.

In addition to the IRF/ISRE TFBS already mentioned above, our study also identified several other TFBSs that might be involved in the immune and inflammatory response. In particular, we found several genes with 5'UR binding-sites for transcription factors that have not yet been implicated in immunity. For example, some genes with a TATA box were upregulated by LPS. Unfortunately, we cannot decipher this "regulatory code" in mammals with current bioinformatics means, although it might be conserved over quite large phylogenetic distances [31]. We also observed five known and one unknown TFBS to be correlated with induction differences between Wt and Tyk2-/- cells. While further analysis will be required to examine the effect of these motifs, we generally observed similar GO classes to be upregulated after six hours of LPS as Nilsson et al. [32] who studied the expression of genes and gene regulation *via *5'UR TFBS in BMDMs after seven hours of LPS stimulation.

Finally, we related our results to information on putative 3'UTR elements. We found many more genes per 3'UTR element than per 5'UR TFBS, possibly because the 3'UTR is evolutionarily more conserved than the 5'UR or because regulation at the 3'UTR is more pleiotropic than at the 5'UR. Overall, our results provide strong evidence for the involvement of 3'UTR elements in the LPS response and in the differential regulation of expression between genotypes. Several classes of conserved 3'UTR regulatory sequences and 3'UTR miRNA cognate sequences were differentially regulated between genotypes, in response to LPS, and in terms of LPS inducibility between Wt and Tyk2 mutant cells. Most notably, LPS strongly induced expression of genes with AU-rich elements (m2/ARE), which have already previously been implicated in innate immunity [33,34]. In contrast, among the miRNA cognate motifs that were differentially regulated in our experiment, none has a known function. Thus, since 3'UTR conserved elements and miRNA consensus sites are still poorly annotated and characterized, it is difficult to biologically interpret these results. Nevertheless, it has become clear in recent years that 3'UTR elements and miRNA cognate sequences are highly important in regulating gene expression, and it will therefore be interesting to explore their potential role in immune and inflammatory transcription.

## Conclusions

We conclude that Tyk2 function is required for the full transcriptional response upon LPS challenge, but dispensable for the LPS induction of IFN-responsive genes. Although it remains presently unclear why Tyk2 mainly mediates baseline but not LPS-induced type I IFN signaling, we suggest that Tyk2 maintains IFN-responsive genes in a primed, "ready to go" state, with factors other than Tyk2 mediating their induction upon immune challenge. Moreover, we conclude that Tyk2 plays a major but previously unknown role in the regulation of metabolism, especially lipid metabolism. Our data suggest that Tyk2 is not only critically required for the downregulation of baseline expression of many metabolic genes, but also for their further LPS-mediated suppression. While these results deserve in-depth future analysis, they clearly strengthen the previously made case for intricate connections between IFN signaling and metabolism and provide evidence for a role of Tyk2 in this connection. A better understanding of the relationship between immunity and metabolism will also likely be important for improving therapeutic interventions that target immune diseases as well as metabolic disorders.

## Methods

### Mice and macrophages

C57BL/6 wildtype (Wt) mice (*Mus musculus*) were purchased from Charles River Laboratories International, Inc. (Wilmington, MA, USA). Tyk2-/- mice are described in [10] and were backcrossed for ten generations into the C57BL/6 background. Mice were housed and bred under specific pathogen free conditions according to FELASA guidelines. All animal experiments were discussed with and approved by the institutional ethics committee and were carried out in accordance with protocols approved by Austrian laws (GZ 68.205/67-BrGT/2003; GZ 68.205/0204-C/GT/2007) and European directives.

Thioglycolate-elicited peritoneal macrophages were isolated from sex- and age-matched mice (males, 8-10 weeks old) as described in [35]. Cells from 2-3 mice per genotype were pooled for each of the three independent replicate experiments. 4 × 10^6 ^cells were plated out on 6 cm cell culture dishes (BD Falcon) and grown under standard conditions (37°C, 5% CO_2_) in DMEM medium (Invitrogen) supplemented with 5% fetal calf serum (FCS), 100 mg/mL penicillin, 100 U/mL streptomycin, 2 mM L-glutamine and 50 μM β-mercaptoethanol. The day after isolation, cells were stimulated with 100 ng/mL LPS (*E. coli *serotype 055:B5, Sigma) or medium only (control), respectively, for six hours. For each combination of genotype (Wt versus Tyk2-/-) and treatment (control versus LPS) we carried out three independent replicate experiments.

### Microarrays and RT-qPCR

For microarrays and RT-qPCR, total RNA was isolated using TRIZOL reagent (Invitrogen) according to the manufacturer's instructions. RNA concentration and integrity were determined by capillary electrophoresis (Agilent Technologies).

Total RNA (2 μg) was reverse transcribed using the iScript First Strand cDNA Synthesis Kit (Bio-Rad) and hybridized to the CodeLink Mouse Whole Genome Bioarray http://www.appliedmicroarrays.com/ according to the manufacturer's protocols. Arrays were scanned with a GenePix Array Scanner 4000B and GenePix Pro 4.0 software (Axon Instruments, Inc.). Data were prepared for statistical analysis (see below) with CodeLink Expression Analysis software http://www.appliedmicroarrays.com/. A gene was recorded as expressed if its unnormalized fluorescence intensity value exceeded 25 (see below). For these expressed genes, we log-transformed their intensity values and normalized them, separately for each array, to a mean of zero and a variance of one. Genes with missing annotation and duplicate gene entries were removed before analysis;7546 genes of the approximately 30,000 genes on the microarray remained after filtering. The microarray dataset has been deposited at Gene Expression Omnibus (GEO; http://www.ncbi.nlm.nih.gov/geo/ with accession number GSE19733).

To validate the expression data from the microarrays we performed reverse transcription-quantitative polymerase chain reactions (RT-qPCR) for about 170 genes including several IFN-inducible genes (Additional File 1). RNA (3 μg/60 μl) was reverse transcribed using the iScript First Strand cDNA Synthesis Kit (Bio-Rad). Taqman qPCR Low Density Custom Arrays (Applied Biosystems) were used to validate the microarray data, either in 48 well (samples in duplicates) or in 96 well (single samples) format. 5 μl cDNA (corresponding to 250 ng input RNA) were used in a 100 μl mastermix per sample-loading port, containing 5 mM MgCl_2_, 200 μM of each dNTP (MBI Fermentas), 1 × ROX reference dye (Invitrogen), 4 Units HotFire DNA polymerase (Solis Biodyne), and 1 × reaction buffer B (Solis Biodyne). RT-qPCR was performed on a ABI PRISM 7900 HT machine (Applied Biosystems) using the following cycling conditions: initial denaturation at 95°C for 12 min, 40 cycles of 95°C for 15 sec and 60°C for 1 min.

Validation of selected addional genes (mixed GO annotations) and genes involved in lipid metabolism was performed with single RT-qPCR assays. RNA (1 μg/20 μl) was reverse transcribed as described above. For assays using probes, we used 0.5 -2 μl cDNA (dependent on the gene analyzed) in a 25 μL mastermix, containing 2.5 mM MgCl_2_, 200 μM of each dNTP (MBI Fermentas), 300 nM primer, 100 nM probe, 1 Unit HotFire DNA polymerase (Solis Biodyne), and 1 × reaction buffer B (Solis Biodyne). For Qiagen assays, we used 0.5-2 μl cDNA in a 25 μL mastermix, containing 2.5 mM MgCl_2_, 200 μM of each dNTP (MBI Fermentas), 1 × QuantiTect primer assay (Qiagen), 0.2 × EvaGreen (Biotium), 1 Unit HotFire DNA polymerase (Solis Biodyne), and 1 × reaction buffer B (Solis Biodyne). RT-qPCR was performed on a Mastercycler realplex (Eppendorf) machine using the following cycling conditions: initial denaturation at 95°C for 15 min, 40 cycles of 95°C for 20 sec and 60°C for 1 min. For Qiagen assays, melting curve analyses were performed in order to check specificity. For details on RT-qPCR assays see Additional File 1.

The resulting data were analyzed using SDS 2.2 (Applied Biosystems) or realplex (Eppendorf) software and the statistical package R [36]. To compare microarray and RT-qPCR data we used log-transformed fluorescence intensity data. To standardize the RT-qPCR data we subtracted the mean Ct values of two endogenous control genes, ubiquitin-conjugating enzyme E2D 2 (UBE2D2) and hypoxanthine phosphoribosyltransferase (HPRT), from the RT-qPCR Ct values of the focal genes analyzed by Taqman qPCR Low Density Custom Arrays. For single RT-qPCR assays, the standard curve method was used to calculate the log expression levels, and values for the endogenous control gene UBE2D2 were subtracted from those of the focal genes. Thus, the microarray and RT-qPCR values were expected to be approximately proportional or inversely proportional, respectively, to the log of the mRNA concentrations of the focal genes. We found a good, approximately linear relationship between log microarray fluorescence intensities and normalized RT-qPCR delta Ct values for medium to highly expressed genes, while at lower levels we generally observed overwhelming background noise (Additional File 1). Importantly, we found a good agreement in terms of approximate fold induction between array and RT-qPCR data in general, and especially for IFN-inducible genes, genes involved in lipid metabolism, and five other genes (Additional File 1).

In order to check the experiment used for microarray analysis for effective LPS stimulation and for the expected differences between Wt and Tyk2-/- cells, IFNβ mRNA expression was monitored with RT-qPCR (data not shown). IRF7 and IRF1 were included in the validation experiments (Additional File 1). mRNA expression patterns of all three genes were as expected [12]. In addition, microarray results for TNFα and NOS2 expression confirmed our previous findings [10,12].

### Analysis of single genes and gene ontology

To analyze the effects of genotype and LPS treatment on gene expression we used Analysis of Variance (ANOVA). For each gene, log-transformed normalized data were used in an univariate fully factorial two-way ANOVA, with genotype (Wt versus Tyk2-/-) and LPS treatment (untreated control versus LPS) as fixed factors:

where *y*_*gti *_is the *gti*-th expression level for each gene, μ is the overall mean, *g *denotes the effect of genotype, *t *the effect of LPS treatment, *gt *the effect of the interaction between genotype and treatment, and *e *the residual error variance. Individual entered the analysis as a random effect.

Data inspection showed that the distribution of variances approximately follows the expectation (assuming all genes have equal variances), i.e. a scaled χ^2^-distribution with four degrees of freedom. Since averaging over all genes gives a much better estimate of residual variation than single gene estimates of error variance, we estimated the effect sizes for each of the three effects (genotype, treatment, and genotype × treatment) as standardized coefficients for each gene, i.e., by dividing the effects by the average of the square root of the residual variance over all genes. This approach is similar to the use of fold changes instead of *t*-values (see [37,38], but see [39] for a critique). To test the robustness of our method we also used moderated *t*-statistics [40]. Since the outcomes of both types of analysis were qualitatively and quantitatively similar (data not shown), we only report the former analysis.

Of the 7546 genes analyzed, 7546 × 0.05 ≈ 377 genes are expected to be significantly different by chance alone, about half of them up and half of them down (assuming a type I error rate or statistical significance level of α = 0.05). To address this multiple testing problem, we used a false discovery rate (FDR) of 0.05 to detect genes that are likely to be differentially regulated between genotypes, influenced by LPS treatment, or both. Results based on genes lists assuming α = 0.01 did not change the qualitative outcome of our analyses (data not shown).

To refine our single gene analysis in terms of biological function we performed gene ontology (GO) analysis, using the classification developed by the GO Consortium http://www.geneontology.org/. This classification consists of three structured controlled vocabularies (or ontologies) that describe gene products in terms of their associated "biological processes", "cellular components", or "molecular functions", in a species-independent manner. We restricted our analysis to the "biological process" annotation, as this category is easiest to interpret and since we observed substantial overlap between this group of genes and the "molecular function" category. For GO analysis within the "biological process" category, genes were ordered into GO classes. We separately contrasted each class with all other classes by performing *t-*tests (assuming normality of the data) and nonparametric Kolmogorov-Smirnov tests (relaxing the assumption of normality) on standardized coefficients. Since data were approximately normally distributed, results differed little between the tests; we therefore only report *t*-tests.

### Analysis of putative regulatory elements

To relate the gene expression effects of genotype and LPS treatment to putative regulatory regions, we used bioinformatic analysis to identify putative *cis*-regulatory elements in the 5'UR, i.e. TFBSs in the promoter region, and 3'UTR regulatory elements (including miRNA targets). We aimed to use the same alignment methods and similar methods for defining these different *cis*-regulatory regions.

Sequence information was obtained from the University of California Santa Cruz (UCSC) Genome Bioinformatics site http://genome.ucsc.edu; in particular, the mouse mm7-assembly and the human hg18-assembly data were used. For each gene, we extracted the region up to 8 kb upstream from the transcription start point (5'UR) and the 3'UTR, using the UCSC KnownGenes database, which has a higher coverage than the RefSeq database. For the few protein coding genes with multiple entries, we randomly kept a single entry. If an adjacent gene was closer than 8 kb in the UR, the recorded upstream sequence was truncated before the start of the transcribed region of the adjacent gene. We also performed our analyses with shorter regions and obtained similar results (data not shown).

Homologous human and mouse genes were acquired using the HomoloGene database http://www.ncbi.nlm.nih.gov/homologene (48.1) at the National Center for Biotechnology Information (NCBI). Only genes with reciprocal best BLAST hit e-values were selected. For each gene, we aligned the 5'UR and the 3'UTR separately using LAGAN [41]. To obtain a reasonable alignment of the 5'UR, we truncated the longer sequence so that it had the same distance from the presumed transcription start site as that of the shorter sequence, despite the fact that in one or the other species the 5'UR might be shortened by another gene. This truncation thus only affected the most upstream part of the region. For orthologous 3'UTRs, we allowed for different lengths because of the generally higher similarity and thus easier alignment. We validated this method extensively (results not shown, see B. Fuhrmann, unpublished diploma thesis) [42]. In particular, we found that pairwise alignments with LAGAN performed better than available multi-species alignments, because fewer regions were excluded and because placement of insertions and deletions in multi-species alignments is problematic.

To identify putative regulatory elements (TFBSs and 3'UTR regulatory sequences) we used a comparative genomics approach. Xie et al. [14] inferred conserved motifs in the 5'UR (putative TFBSs) and 3'UTR (containing many miRNA cognate sequences) using a comparison among mammalian genomes. While we are aware that regulatory sequences may lie outside of these regions (e.g., in the 5' untranslated region (5'UTR) or in introns, especially the first), we refrained from including such regions, for two reasons. First, as a transcribed region, the 5'UTR imposes more (and different) constraints on regulatory sequences than the untranscribed 5'UR that are difficult to account for. Second, the search space would have increased vastly with the inclusion of introns, which also would have caused alignment problems. We note that the list of Xie et al. [14] contains several unknown putative TFBSs, whereas some known TFBSs are not included. Furthermore, Xie et al. [14] do not distinguish between TFBSs of some related transcription factors (e.g., ISGF3 and most other IRF TFBSs). We used these putative regulatory sequences as raw data for our analysis. We parsed each gene of the aligned human and mouse sequences for conserved motifs in the lists given in Xie et al. [14]: for putative TFBSs see Supplementary Material Table S3, for putative 3'UTR regulatory sequences Supplementary Material Table S4, and for conserved octamer motifs in the 3'UTR Supplementary Material Table S5, respectively. To exclude false positives, a motif was defined as conserved if it occurred in the homologous mouse and human sequence at the same position of the alignment in any of its forms (including the reverse complement for the 5'UR). To validate the ability of this comparative genomics approach to identify 5'UR TFBSs we used publicly available microarray data http://symatlas.gnf.org/SymAtlas/ on samples from diverse mouse tissues (see Additional File 9). We noticed some discrepancies between the annotation of Xie et al. [14] and the list of miRNAs in miRBase http://www.mirbase.org/[15-17]. Conserved octameric sequences in [14] were therefore re-annotated using the list in miRBase release 10.1. An association was called if the distal octamer of the mature miRNA matched perfectly to the octameric region.

To analyze TFBSs and 3'UTR regulatory elements, we contrasted the normalized effect coefficients of genes that had at least one conserved regulatory element with those of the rest of the genes. As for the GO analysis, we performed both *t*-tests Kolmogorov-Smirnov tests. Since the outcome of these tests was again qualitatively similar, we only report *t*-tests.

For our TFBS analysis (see above) we used a rather stringent definition [14], filtered out all non-conserved and non-alignable motifs, and excluded all weakly expressed genes, such that our analysis was based on only 40 genes containing an IRF/ISRE TFBS, with many known IFN-inducible genes missing. To extend this analysis and to explore different inclusion criteria for 5'UR TFBSs, we produced a list of 187 known IFN-responsive genes from the literature (without differentiating between type I IFN and IFNγ-responsive genes) and asked how many of these genes show expression changes in our experiment. As above, we analyzed the standardized effects of genotype, treatment, and genotype × treatment on gene expression (Additional File 3, Table 1). Furthermore, we compared IFN-responsive genes to all other genes with respect to IRF/ISRE or GAS TFBSs in their 5'UR. We used different criteria for including or excluding TFBSs, i.e. we report enrichments for conserved and nonconserved TFBSs that differ in the length of the upstream search region (Additional File 3, Table 2). We also lowered the specificity of our screening for TFBSs of known IFN-induced transcription factors by allowing for mismatches (data not shown).

## Abbreviations

FDR: false discovery rate; IFN: interferon; IL: interleukin; IRF/ISRE: IFN-responsive factor/IFN-stimulated response element; ISGF3: interferon stimulated gene factor 3; JAK/STAT: Janus kinase/signal transducer and activator of transcription; LPS: lipopolysaccharide; miRNA: microRNA; TFBS: transcription factor binding-site; RT-qPCR: reverse transcription-quantitative polymerase chain reaction; 5'UR: 5' untranslated region upstream of transcription start site of protein coding genes; 3' UTR: 3' untranslated region of protein coding genes; Wt: wildtype.

## Authors' contributions

MM, BS, and PK designed and supervised experiments; EH, BW, and RS performed experiments; CV supervised and carried out statistical and bioinformatic analyses, BF conducted bioinformatic analyses; MM, BS, BW, EH, PK, and CV wrote the initial version of the manuscript; TF, CV, BS, and MM rewrote and revised the manuscript. All authors read and approved the final manuscript.

## Supplementary Material

Additional file 1 **RT-qPCR validation of microarray data**. This file contains independent validation of the microarray expression data with RT-qPCR, with a particular emphasis on IFN-responsive genes and genes involved in lipid metabolism. Click here for file

Additional file 2 **Analysis of genes with putative TFBS**. This file contains a list of gene classes grouped according to their putative 5'UR TFBS and a t-test analysis of their expression patterns. Click here for file

Additional file 3 **Analysis of IFN-inducible genes**. This file contains a list of IFN-inducible genes and a t-test analysis of their expression patterns. Click here for file

Additional file 4 **Analysis of 3'UTR regulatory elements**. This file contains a list of gene classes grouped according to putative 3'UTR regulatory sequence and a t-test analysis of their expression patterns. Click here for file

Additional file 5 **Expression of genes with the m2/ARE 3'UTR regulatory sequence**. This file contains a plot of the effects on expression levels of genes containing the m2/ARE 3'UTR regulatory sequence. Click here for file

Additional file 6 **Analysis of 3'UTR miRNA cognate motifs**. This file contains a list of genes with 3'UTR miRNA cognate sequences and a t-test analysis of their expression patterns. Click here for file

Additional file 7 **Comparison to microarray data of Thomas et al**. [20]. This file contains a graph depicting the relationship between the effects on expression of genes examined by Thomas et al. [20] and our study. Click here for file

Additional file 8 **Comparison to microarray data of Fleetwood et al**. [21]. This file contains a graph depicting the relationship between the effects on expression of genes examined by Fleetwood et al. [21] and our study. Click here for file

Additional file 9 **Validation of comparative approach to identify TFBS**. This file contains a bioinformatic validation of the comparative approach to identify 5'UR TFBSs using publicly available microarray gene expression data. Click here for file
