# Supplementary material for: Transcriptome analysis reveals a major impact of JAK protein tyrosine kinase 2 (Tyk2) on the expression of interferon-responsive and metabolic genes
Source: BMC Genomics. 2010 Mar 25;11:199. doi: 10.1186/1471-2164-11-199 (PMC2864243; doi:10.1186/1471-2164-11-199)
Supplement: Additional file 1 — RT-qPCR validation of microarray data. This file contains independent validation of the microarray expression data with RT-qPCR, with a particular emphasis on IFN-responsive genes and genes involved in lipid metabolism. [file 1471-2164-11-199-S1.PDF]

Additional File 1

Comparison microarray versus RT-qPCR

Figure 1

Comparison of about 100 genes between microarray and Taqman RT-qPCR; technical quality

Expression levels were determined with RT-qPCR using the same cDNA as was used for the microarrays. x-axis: relative expression levels (delta Ct levels compared to two housekeeping genes) from RT-qPCR; y-axis: log-expression levels (as log of the fluorescence intensities plus one) from microarrays. Genes were selected prior to the microarray experiments, in part because of their potential involvement in innate immunity. No gene was removed when displaying low fluorescence intensity with the microarray or a high Ct with RT-qPCR. Each gene is represented by three replicate samples in two genotypes and two treatments. To the left of the vertical line (at a Taqman delta Ct level of 3), the correlation is -0.56, but to the right the correlation is only -0.21, indicating a low reliability for weakly expressed genes due to overwhelming background noise. The regression line is calculated only for the part of the dataset to the left of the vertical line.

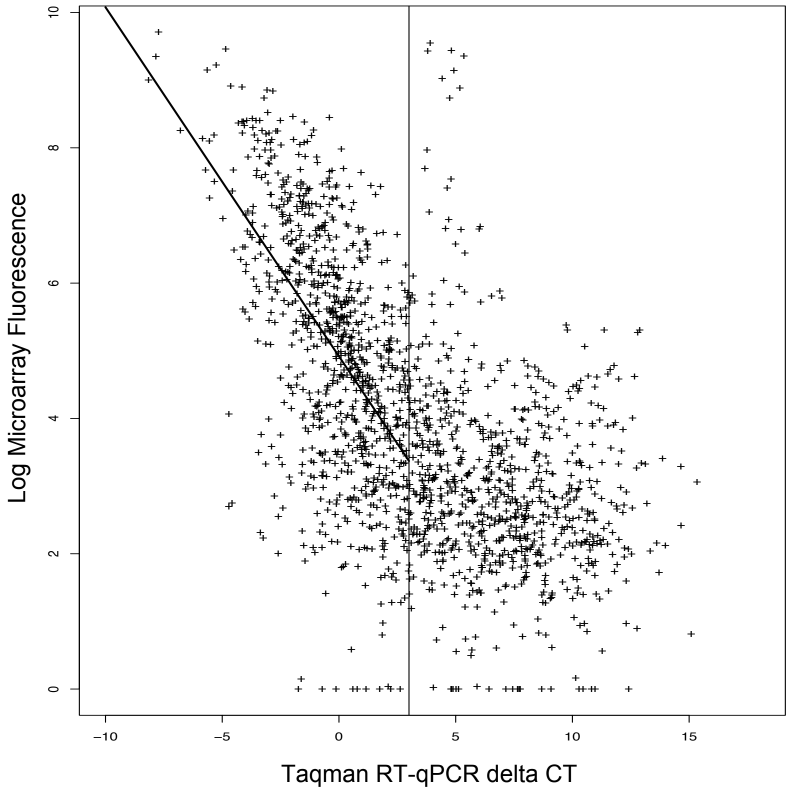

Figure 2

Expression profiles of immune related and lipid metabolism annotated genes.

Expression levels of nine genes in Wt and Tyk2-/- (Tk) peritoneal macrophages either at the basal level (0) or after six hours of LPS treatment (6), as determined by RT-qPCR, are shown. The first four genes (CASP8, CCL7, IL15, KITL) are annotated for involvement in the immune response, the other five genes (SDC2, CRAT, FADS1, FASN, MVD) for lipid metabolism. The genes were chosen in order to validate specific expression patterns observed in the microarray. Expression levels are shown relative to the mean basal level in the Wt, error bars are plus/minus the standard error.

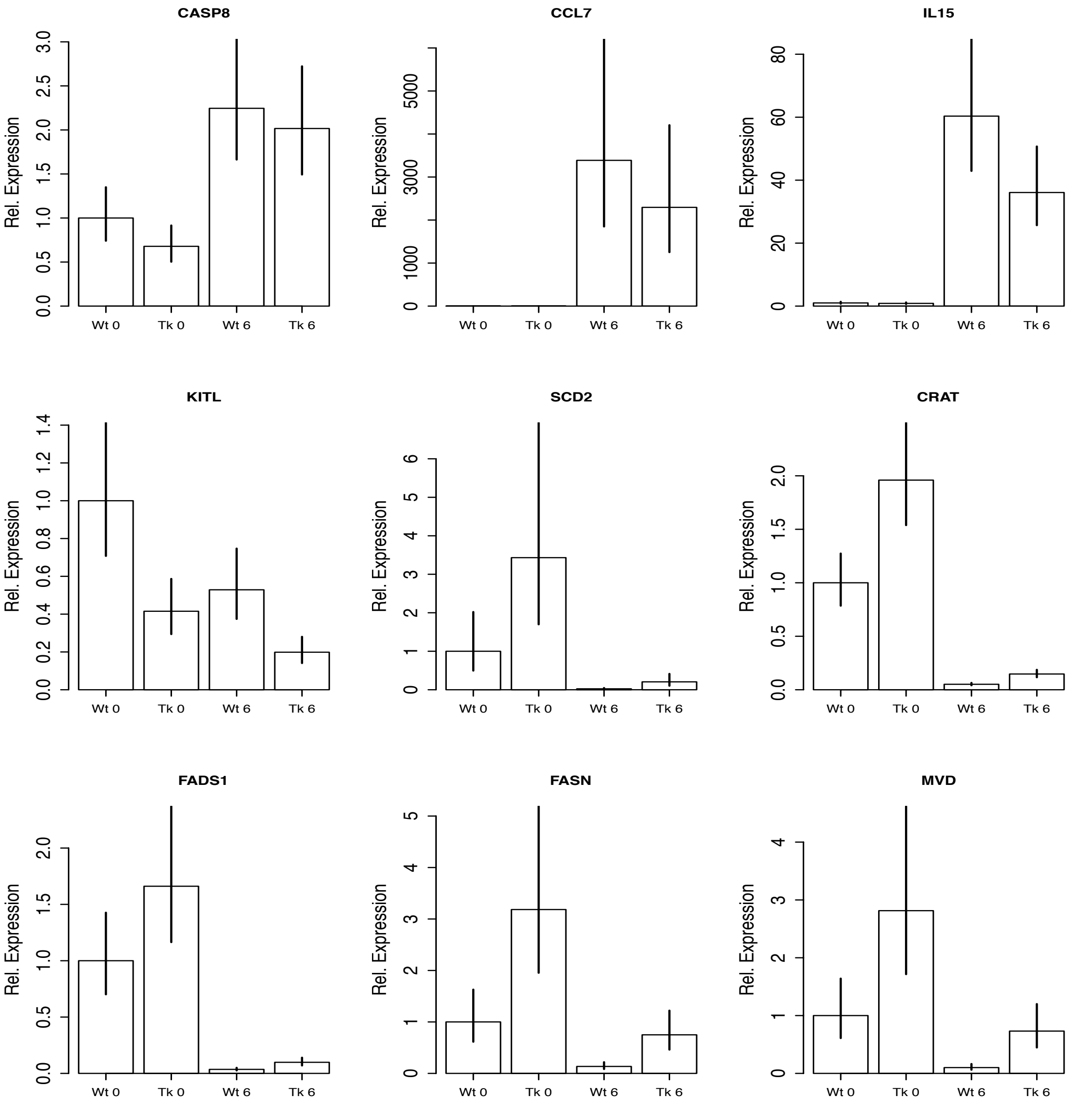

Table 1

Comparison of expression ratios between microarray and RT-qPCR, mainly immune annotated genes

Gene	Assay ID	expression ratios microarray gt	lps	int	expression ratios RT-qPCR gt	lps	int
**A)**							
FGR	Mm00438949_m1	0.94	0.47*	0.83*	0.87	0.25*	0.39*
CSNK1A1	Mm00521599_m1	1.04	0.99	0.98	1.31	1.01	0.78
SKIV2L	Mm00451675_m1	0.95	1.11	1.02	0.99	1.49*	1.44
EIF2C1	Mm00462977_m1	0.90	0.73*	0.85	0.74	0.89	0.83
SCYE1	Mm00433034_m1	0.94	0.99	1.04	1.09	1.07	0.98
NFKBIB	Mm00456849_m1	1.11	2.08*	1.05	1.36	5.49*	1.87*
CASP2	Mm00432314_m1	1.24*	1.26*	1.19*	1.08	1.35	2.16*
DAB2	Mm00517751_m1	0.80*	2.31*	1.25*	0.78	1.96*	0.96
KHDRBS1	Mm00516130_m1	0.94	1.15	1.14	0.75	2.05*	2.20*
MITF	Mm00434954_m1	0.91	1.01	0.91	0.59*	1.29	1.25
AS3MT	Mm00491075_m1	0.76*	0.56*	0.92	0.79	0.30*	0.56*
RPTOR	Mm00712676_m1	1.12	1.51*	1.02	0.84	1.13	0.84
FMR1	Mm00484415_m1	1.28*	1.91*	1.21*	1.13	2.84*	2.12*
ICSBP1	Mm00492567_m1	1.27*	1.57*	1.59	1.04	2.24*	5.44*
**CCL5**	**Mm01302428_m1**	**1.45***	**5.23***	**1.12**	**2.59**	**19.80***	**2.11***
NEDD4	Mm00456829_m1	1.02	1.40*	0.98	0.71	0.62*	0.87
MDM4	Mm00484944_m1	1.03	1.48*	1.08	1.00	2.32*	1.15
PRDX4	Mm00450261_m1	1.00	0.90	1.03	0.88	1.18	1.30
PFTK1	Mm00448111_m1	1.12	0.96	1.20*	1.42	1.86*	2.37*
AIF1	Mm00479862_g1	3.17*	0.60*	1.21*	5.35*	0.60*	3.11*
ARG2	Mm00477592_m1	1.01	1.99*	0.94	1.00	1.99*	0.87
CD84	Mm00488934_m1	0.99	0.69*	0.93	1.10	0.49*	0.85
BIRC1B	Mm00440446_m1	0.95	1.06	0.96	1.03	1.09	0.82
DDX50	Mm00459758_m1	1.06	0.74*	0.91	0.84	0.56*	0.89
TRAF1	Mm00493827_m1	1.37*	4.84*	0.93	1.21	10.04*	1.42
RALY	Mm00499167_m1	1.13	0.93	1.03	0.67*	1.07	1.48
PYCARD	Mm00445747_g1	1.07	0.62*	1.01	1.18	0.50*	1.08
HMBS	Mm00660262_g1	0.93	0.40*	0.83*	0.99	0.23*	0.38*
KLF2	Mm00500486_g1	1.27*	0.43*	0.98	1.29	0.34*	0.98
**STAT1**	**Mm00439518_m1**	**3.92***	**2.21***	**0.82***	**5.52***	**3.67***	**1.17**
PRDX6	Mm00725435_s1	0.75*	0.81*	0.94	0.54*	1.03	0.85
IQGAP1	Mm00443860_m1	1.12	1.00	1.00	0.85	1.47*	1.38
TNFRSF26	Mm00558700_m1	0.88	0.59*	0.98	0.92	0.59*	0.91
IRF7	Mm00516788_m1	15.58*	1.99*	0.49*	93.94*	5.28*	0.27*
CD79A	Mm00432423_m1	1.09	1.14	0.91	2.81*	0.69*	0.86
ZFP291	Mm00615854_m1	0.97	0.76*	1.12	0.85	0.79	1.33
MGA	Mm00465485_m1	0.91	0.96	0.95	0.96	0.52*	0.54*
CIAS1	Mm00840904_m1	1.48*	5.70*	0.88	1.58*	13.03*	1.22
NR2F6	Mm00438762_m1	0.95	0.34*	0.77*	0.77	0.35*	0.59
ERCC4	Mm00516619_m1	0.95	0.81*	0.90	1.04	0.70	0.68
HIPK2	Mm00439329_m1	0.83	1.04	1.02	0.71	1.38	1.28
CSNK1G1	Mm00557447_m1	0.82*	1.42*	1.09	0.95	1.14	1.04

PADI4	Mm00478087_m1	1.59*	0.65*	1.00	1.74*	0.51*	1.53
BCAR3	Mm00600213_m1	1.03	1.08	1.31*	1.30	1.34	2.20*
SRRM1	Mm00489728_m1	0.97	1.53*	1.09	0.87	1.52*	1.60
**DDX58**	**Mm00554529_m1**	**2.60***	**5.54***	**1.05**	**3.10***	**8.17***	**1.61**
DNMT1	Mm00599763_m1	0.94	0.40*	0.87*	0.89	0.31*	0.69
EEF1E1	Mm00470535_m1	1.18	1.28*	0.83	1.01	1.76*	0.80
JAK1	Mm00600614_m1	0.89	1.03	1.04	0.94	1.57*	1.73*
RAC2	Mm00485472_m1	0.86	1.11	1.01	0.81	1.38	1.12
GBP2	Mm00494575_m1	3.58	4.79*	0.88	8.30*	9.09*	1.03
LPIN2	Mm00522390_m1	1.03	1.73*	1.12	1.12	0.88	1.07
FER1L3	Mm00621780_m1	0.89	1.04	1.10	0.65*	1.02	1.15
**IRF1**	**Mm00515191_m1**	**3.21***	**1.83***	**0.90**	**3.51***	**2.45***	**1.19**
SUPT6H	Mm00486479_m1	0.96	1.06	0.89	0.79	1.44	1.31
**B)**							
**IFIT1**	**Mm00515153_m1**	**11.87-**	**11.48-**	**0.35-**	**22.84***	**21.62***	**0.45**
**OAS1B**	**Mm00449297_m1**	**3.04-**	**3.55-**	**0.97-**	**3.81***	**7.57***	**1.56**
**SOCS1**	**Mm00782550_s1**	**1.92-**	**9.20-**	**0.70-**	**2.51***	**22.06***	**0.59**

Comparison between microarray and RT-qPCR for the same genes as in Fig.1 above. Genes were selected prior to the microarray experiments, in part because of their potential involvement in innate immunity. gt: difference between genotypes at the basal level; lps: induction following LPS treatment in the Wt; int: difference in LPS induction between genotypes. Significant tests (at a level of $\alpha = 0.05$) are indicated with an asterisk. Genes known to be IFN-responsive are shown in boldface. ICSBP1 corresponds to the gene IRF8. For part A) genes with low expression values either in the microarray or the RT-qPCR were filtered out. The IFN-responsive genes in part B) showed low expression in the basal untreated state in the microarray experiment, such that no tests could be performed for the microarray. Taqman assay ID’s are given after the gene symbol. "–" indicates that the test could not be calculated because of too low expression levels.

Table 2

Comparison of expression ratios between microarray and RT-qPCR, lipid and mixed annotated genes

	expression ratios microarray			expression ratios RT-qPCR		
**Gene**	**gt**	**lps**	**int**	**gt**	**lps**	**int**
**A)**						
FASN	0.59*	0.61*	0.71*	0.45*	0.37*	0.68
FADS1	0.73*	0.37*	0.52*	0.70	0.14*	0.70
CRAT	0.89	0.31*	0.56*	0.63*	0.17*	0.77
MVD	0.61*	0.35*	0.42*	0.49	0.39*	0.52
SCD2	0.52*	0.25*	0.35*	0.43	0.14*	0.53
**B)**						
GADD45B	1.18	3.47*	1.19*	0.76	4.07*	0.65
TLK2	1.16	3.14*	1.43*	0.90	1.96	0.34*
MOV10	2.23*	4.20*	1.27*	0.24	1.02	0.50
ZBP1	5.23*	2.53*	0.74*	0.06*	5.05*	0.16*
HDAC5	0.97	0.22	0.77*	1.37	0.36	1.02

Comparison between microarray and RT-qPCR. The RNA was from different biological replicates, i.e. variation between microarray and RT-qPCR reflects both biological and technical variation. Single gene RT-qPCR and standardization with an endogenous control gene was used. Part A) shows genes involved in lipid metabolism (selected for validation of the microarray because of their known involvement in lipid metabolism), part B) genes with mixed annotation that showed a significant interaction NC in the microarray experiment. Approximate expression ratios were measured using either microarrays or RT-qPCR. gt: difference between genotypes at the basal level; lps: expression ratio of the LPS induction in the Wt; int: expression ratio (genotype x treatment). "∗" indicates significance ($p < 0.05$).

Assays, primers and probes used

Assays for UBE2D2 and IFNβ were performed as described in the Real-Time PCR Primer and Probe Database (http://medgen.ugent.be/rtprimerdb/; RTPrimerDB ID 3377 and 3378, respectively). Assays for FADS1, FASN, MVD, SCD2 and CRAT were from Qiagen (QuantiTect primer assays QT00114184, QT00149240, QT00115976, QT00140434 and QT00111405). Primers and probes for GADD45B, HDAC5, MOV10, TLK2 and ZBP1 were as follows:

GADD45B: CTGCCTCCTGGTCACGAACT (fwd),
GAGATATAGGGGACCCATTGGTTA (rev),
FAM-CTTGGTGGAGGTGGCCAGTTACTGTGAA-BHQ1 (probe);

HDAC5: CGTGGACAGGAGGTGTGGAT (fwd),
GGTCAAGTGGCCAAAACATCTG (rev),
FAM-ACAGTGGTGATGCCCATTGCCCA-BHQ1 (probe);

MOV10: CCGTACCGGAAGCAGGTAGA (fwd),
CACAGAGCCCACCTTCAAATCT (rev),
FAM-AATCCGTTACTGCATCACAAAACTTGACCG-BHQ1 (probe);

TLK2: CCGCCAAAGCCAGTAGTAACA (fwd),
CTTTCGGATGTGAGGCAACA (rev),
FAM-CAAAGGCATTTATCAGGAGATGTCTGGCCTATC-BHQ1 (probe);

ZBP1: AAGAAGGAAGACACAGGTACAAGTC (fwd),
GTGGGCAGCATGGAGTCACT (rev),
FAM-CACTCCACCCAGAAGCTGCCAGCA-BHQ1 (probe).
