# Supplementary material for: Transcriptome analysis reveals a major impact of JAK protein tyrosine kinase 2 (Tyk2) on the expression of interferon-responsive and metabolic genes
Source: BMC Genomics. 2010 Mar 25;11:199. doi: 10.1186/1471-2164-11-199 (PMC2864243; doi:10.1186/1471-2164-11-199)
Supplement: Additional file 4 — Analysis of 3'UTR regulatory elements. This file contains a list of gene classes grouped according to putative 3'UTR regulatory sequence and a t-test analysis of their expression patterns. [file 1471-2164-11-199-S4.PDF]

Additional File 4

Analysis of 3'UTR regulatory elements

motifnr	# genes	mean_gt	p_gt	mean_lps	p_lps	mean_int	p_int
m43	254	-0.33	0.0009	-0.10	0.1978	0.02	0.3251
m11	623	-0.23	0.0026	-0.13	0.1301	-0.02	0.3243
m57	110	0.39	0.0037	0.02	0.3547	-0.10	0.8820
o7	207	-0.33	0.0058	-0.34	0.9720	-0.05	0.7941
o38	142	-0.31	0.0174	0.38	0.0063	0.12	0.0693
o62	77	-0.43	0.0176	-0.07	0.4765	-0.05	0.8704
o61	7	-1.02	0.0208	-2.12	0.3589	-0.47	0.5697
o67	4	-0.46	0.0212	0.05	0.8329	0.83	0.2881
o40	45	-0.44	0.0226	-0.14	0.6467	0.23	0.1184
m20	262	0.16	0.0255	0.40	0.0024	0.02	0.3035
m36	208	-0.29	0.0315	-0.19	0.5500	-0.03	0.6890
m98	31	-0.44	0.0331	0.63	0.0874	0.22	0.2434
m67	164	0.13	0.0349	0.68	0.0004	0.13	0.1282
m100	70	0.22	0.0428	0.60	0.0079	0.17	0.0856
m38	222	-0.28	0.0469	0.13	0.0411	0.10	0.1057
o55	28	-0.73	0.0530	-0.08	0.6664	-0.09	0.9718
m93	37	-0.44	0.0536	-0.55	0.7557	-0.10	0.9373
o18	278	0.09	0.0552	0.65	0.0001	0.15	0.0301
o45	8	-1.08	0.0554	-1.26	0.5672	-0.23	0.5511
m40	235	-0.24	0.0604	0.13	0.0697	0.11	0.0548
o47	61	-0.33	0.0624	-0.01	0.4424	0.08	0.3997
o11	289	-0.22	0.0638	0.04	0.1044	0.08	0.0878
o66	19	-0.62	0.0660	0.06	0.6795	-0.21	0.7571
m31	347	-0.20	0.0758	0.21	0.0018	0.00	0.2989
o37	6	1.02	0.0780	2.03	0.3950	1.62	0.2366
o20	148	-0.25	0.0853	-0.08	0.3360	-0.03	0.6683
o9	136	-0.28	0.0897	0.07	0.2379	-0.04	0.8164
m81	61	0.19	0.0910	-0.36	0.9839	-0.27	0.2680
o39	6	-0.44	0.1169	0.59	0.0821	0.15	0.4067
m105	6	-0.44	0.1169	0.59	0.0821	0.15	0.4067
m74	78	-0.33	0.1196	-0.28	0.8735	0.17	0.2041
o27	6	-0.99	0.1333	-1.12	0.3309	-0.93	0.0924
m26	383	-0.17	0.1348	0.15	0.0017	0.02	0.1825
m22	332	-0.18	0.1429	-0.07	0.1636	0.06	0.0915
m86	70	-0.27	0.1436	0.50	0.0773	0.11	0.2695
o22	65	-0.35	0.1461	-0.36	0.9785	0.12	0.3258
m34	188	-0.19	0.1647	0.16	0.0391	-0.05	0.7760
m71	24	-0.44	0.1716	0.33	0.4378	0.50	0.1792
m19	308	-0.16	0.1844	0.25	0.0028	0.07	0.1012
m83	54	0.15	0.1855	-0.16	0.7062	0.10	0.3886
m69	40	-0.28	0.1920	0.49	0.1091	-0.04	0.8783
m8	337	-0.17	0.1940	0.17	0.0077	0.13	0.0168
o36	46	-0.25	0.1964	-1.02	0.1414	-0.26	0.3821
m27	194	-0.19	0.2070	0.21	0.0322	0.08	0.1751
m80	69	-0.32	0.2071	-0.27	0.8653	0.15	0.2131
m10	319	-0.18	0.2096	-0.16	0.3681	-0.09	0.8531
o43	142	-0.18	0.2173	0.49	0.0021	0.00	0.5023
m5	1175	-0.12	0.2249	0.06	0.0000	0.02	0.0148
m58	45	-0.40	0.2289	-0.33	0.9530	-0.05	0.8857
m56	46	-0.27	0.2294	-0.77	0.3602	-0.05	0.9070
m88	32	-0.36	0.2297	-0.60	0.6470	0.33	0.0831
m15	158	-0.18	0.2316	0.36	0.0421	-0.02	0.6577

m85	82	0.13	0.2335	0.84	0.0043	0.22	0.1233
m79	67	0.21	0.2375	0.43	0.1101	-0.05	0.8792
m1/TATA-box	1938	-0.11	0.2382	-0.54	0.0091	-0.09	0.7796
o46	17	0.35	0.2388	1.86	0.0226	1.07	0.0066
m60	41	-0.29	0.2493	-0.18	0.7163	-0.13	0.8219
o26	291	-0.17	0.2552	0.11	0.0295	0.10	0.0496
m52	118	-0.21	0.2696	0.37	0.0605	0.12	0.1486
o72	10	0.36	0.2761	-1.01	0.7345	-0.15	0.9150
m37	68	-0.24	0.2974	0.47	0.0241	0.28	0.0298
m73	80	-0.19	0.3004	-0.41	0.8552	-0.14	0.6992
m65	87	-0.20	0.3093	0.32	0.1205	0.17	0.0883
m44	193	0.01	0.3100	0.05	0.1074	0.07	0.1719
m84	70	0.14	0.3201	-0.17	0.6732	-0.31	0.2246
m87	30	-0.29	0.3203	0.57	0.2134	0.01	0.7433
o31	57	-0.21	0.3232	-0.19	0.7487	-0.05	0.8833
o5	191	-0.15	0.3332	0.16	0.0903	-0.10	0.8053
m46	93	-0.27	0.3479	-0.64	0.4253	-0.01	0.6312
o58	6	-0.67	0.3482	0.32	0.6489	-0.18	0.8454
o2	445	-0.14	0.3524	0.01	0.0521	-0.14	0.3720
o14	128	0.05	0.3582	0.47	0.0762	0.10	0.3234
m23	144	0.03	0.3614	-0.08	0.4432	-0.06	0.9144
o71	16	-0.34	0.3801	-0.21	0.7997	0.22	0.3601
o59	13	-0.32	0.3802	0.43	0.2254	-0.03	0.9009
o10	23	0.11	0.3804	0.11	0.5508	0.43	0.1617
m42	274	-0.14	0.3816	0.04	0.0653	0.09	0.0723
m59	33	-0.24	0.3821	0.43	0.1834	0.05	0.6065
o52	7	0.26	0.3857	-2.79	0.3908	-1.52	0.0469
m92	41	-0.29	0.3904	0.82	0.0813	0.35	0.1302
m54	40	-0.22	0.3969	0.37	0.1475	0.24	0.1705
o13	4	0.54	0.3988	4.36	0.2582	1.33	0.4479
m62	34	-0.25	0.4120	-0.14	0.6844	0.06	0.5126
o49	43	-0.21	0.4126	0.02	0.3410	0.10	0.3534
m91	66	0.04	0.4175	0.60	0.0502	0.56	0.0046
m9	942	-0.11	0.4247	0.24	0.0000	0.06	0.0016
m32	202	-0.17	0.4296	0.00	0.2706	-0.03	0.6898
m50	108	-0.22	0.4321	0.64	0.0081	0.26	0.0263
m75	45	-0.25	0.4435	-0.96	0.2202	-0.65	0.0131
o57	21	0.20	0.4460	1.55	0.0366	0.54	0.1478
o68	24	-0.34	0.4515	-1.29	0.3770	-0.45	0.3388
o16	299	-0.12	0.4530	-0.01	0.0953	0.09	0.0628
m55	39	-0.21	0.4625	-0.09	0.6518	-0.05	0.9116
m17	196	-0.14	0.4678	-0.07	0.2856	0.14	0.0636
m89	47	0.03	0.4748	-0.31	0.9446	-0.25	0.4638
o19	36	-0.23	0.4790	0.49	0.1258	0.26	0.2941
o12	196	-0.14	0.4894	0.48	0.0012	0.11	0.0788
m16	213	-0.01	0.4946	0.99	0.0000	0.08	0.1223
o53	64	0.03	0.4955	0.44	0.1050	0.01	0.7021
m101	23	-0.31	0.4959	0.26	0.4928	0.27	0.2743
m30	85	-0.17	0.5147	0.49	0.0133	0.05	0.3545
m103	41	0.05	0.5147	1.05	0.0048	0.30	0.1485
m39	95	-0.15	0.5243	-0.03	0.4487	-0.05	0.8578
m18	244	-0.13	0.5263	-0.26	0.7207	0.05	0.2319
o29	101	-0.17	0.5283	-0.30	0.9032	0.01	0.6007
o33	21	-0.26	0.5430	1.49	0.0430	0.68	0.0524
o60	47	-0.21	0.5438	-0.54	0.6690	-0.25	0.3877
o4	375	-0.12	0.5701	-0.20	0.4294	-0.11	0.6653
o35	94	-0.15	0.5807	-0.25	0.8132	0.11	0.2829
m47	79	-0.15	0.5856	-0.25	0.8010	-0.05	0.8762

m106	13	0.23	0.5945	0.42	0.6261	-0.04	0.9560
o42	13	0.23	0.5945	0.42	0.6261	-0.04	0.9560
m68	92	0.01	0.6313	0.25	0.1266	0.26	0.0558
o50	54	-0.16	0.6313	0.00	0.4107	-0.08	0.9913
m7	348	-0.10	0.6536	-0.13	0.2156	-0.03	0.4980
o3	169	-0.12	0.6629	-0.11	0.4154	-0.02	0.6409
m28	31	0.03	0.6658	-0.52	0.7818	-0.53	0.1856
m53	42	0.03	0.6662	2.60	0.0076	0.17	0.2765
m70	136	-0.12	0.6667	0.48	0.0108	0.21	0.0356
o1	229	-0.11	0.6693	-0.35	0.9873	-0.11	0.7714
m63	50	-0.16	0.6709	-0.84	0.2715	-0.25	0.3741
m96	2	0.49	0.6728	8.98	0.1796	3.89	0.2941
m77	100	-0.13	0.6873	-0.60	0.5077	-0.22	0.3194
o30	30	0.03	0.6978	0.05	0.5062	0.30	0.1797
o32	155	-0.12	0.6993	0.36	0.0067	0.11	0.1227
m61	75	-0.13	0.7049	0.70	0.0634	0.04	0.5265
m94	34	0.00	0.7162	-0.04	0.5889	-0.18	0.6758
o28	191	-0.12	0.7236	-0.11	0.4574	-0.07	0.9784
m45	59	-0.02	0.7277	0.27	0.1405	0.21	0.2007
m12	246	-0.10	0.7287	-0.29	0.8075	-0.14	0.5283
o25	105	-0.03	0.7383	0.86	0.0011	0.12	0.1826
m95	56	-0.02	0.7448	1.03	0.0004	0.43	0.0130
o54	67	0.01	0.7581	0.26	0.1476	-0.07	0.9751
m25	488	-0.09	0.7615	0.21	0.0006	0.00	0.2552
m33	119	-0.10	0.7760	1.04	0.0026	0.03	0.4214
o34	75	-0.12	0.7793	1.22	0.0006	0.39	0.0156
o63	69	-0.03	0.7799	-0.20	0.7481	-0.40	0.0763
m82	69	-0.03	0.7882	-0.30	0.9257	-0.13	0.7770
m76	35	-0.13	0.7884	0.78	0.0538	-0.14	0.7804
m49	41	-0.01	0.7919	-0.51	0.8382	-0.40	0.2207
m13	1015	-0.08	0.7923	0.30	0.0000	0.07	0.0015
o41	2	-0.45	0.7979	1.42	0.2773	0.52	0.5497
o8	114	-0.04	0.8133	0.26	0.0957	0.09	0.1920
o17	64	-0.12	0.8159	-0.29	0.8930	-0.11	0.8299
o21	363	-0.06	0.8257	0.26	0.0011	-0.03	0.5260
o48	14	-0.03	0.8363	-0.46	0.8994	-0.64	0.1000
o51	6	-0.25	0.8468	1.76	0.1577	0.31	0.4212
o64	19	-0.15	0.8506	-0.25	0.8785	-0.03	0.8658
o69	23	-0.12	0.8571	0.02	0.5487	-0.18	0.6942
m97	27	-0.10	0.8901	-0.10	0.7384	-0.14	0.8042
m51	134	-0.09	0.8910	-0.12	0.4684	-0.03	0.7266
o70	15	0.01	0.8922	-0.30	0.9615	0.05	0.8082
m24	332	-0.06	0.9062	0.47	0.0001	0.08	0.0371
m90	67	-0.09	0.9207	-0.33	0.9589	-0.28	0.2241
m3	481	-0.08	0.9222	0.43	0.0000	0.11	0.0056
o15	253	-0.08	0.9257	0.43	0.0011	0.06	0.1007
m72	78	-0.05	0.9296	-0.36	0.9800	-0.36	0.1100
m41	224	-0.06	0.9309	0.36	0.0025	-0.03	0.5915
m66	74	-0.06	0.9322	0.11	0.2208	0.04	0.5188
m29	232	-0.07	0.9346	0.37	0.0013	0.12	0.0425
o6	217	-0.08	0.9417	0.22	0.0320	0.16	0.0359
m2/ARE	675	-0.08	0.9453	0.82	0.0000	0.07	0.0102
m64	47	-0.06	0.9454	-0.03	0.6170	0.27	0.1378
o23	112	-0.08	0.9522	0.59	0.0459	-0.02	0.7122
m14	262	-0.08	0.9574	-0.07	0.2238	-0.05	0.7521
o44	2	-0.01	0.9628	1.29	0.8093	-0.17	0.9586
m35	54	-0.08	0.9725	1.18	0.0026	0.38	0.0263
m48	118	-0.08	0.9785	0.11	0.2039	0.10	0.1613

m6	241	-0.07	0.9841	-0.24	0.6980	-0.25	0.0983
m21	65	-0.07	0.9939	-0.17	0.7719	0.15	0.3507
m4	625	-0.07	0.9946	0.29	0.0000	0.05	0.0324
o65	5	-0.08	0.9953	-0.22	0.9113	-0.32	0.6482

List of gene classes grouped according to their putative 3'UTR regulatory sequence. Abbreviations: motifnr, number of the motif in Xie et al. [14] ; # genes, the number of genes containing the motif; mean, the average difference of the mean normed coefficients of the genes containing the motif between genotypes at the basal level (gt), between WT treated with LPS and untreated WT (lps), or for differential LPS induction between genotypes, i.e., the genotype by treatment interaction (int); p, the p-value of the null hypothesis of no difference in the respective comparison.
