# Supplementary material for: Transcriptome analysis reveals a major impact of JAK protein tyrosine kinase 2 (Tyk2) on the expression of interferon-responsive and metabolic genes
Source: BMC Genomics. 2010 Mar 25;11:199. doi: 10.1186/1471-2164-11-199 (PMC2864243; doi:10.1186/1471-2164-11-199)

Additional File 5

Expression of genes with the m2/ARE 3'UTR regulatory sequence

Effects of (A) Tyk2 genotype (WT minus Tyk2-/-), (B) LPS treatment (6 hours of LPS minus control), and (C) genotype by treatment interaction (difference in LPS induction between WT and Tyk2-/-) on expression levels of genes annotated for metabolism, plotted as normed effect coefficients (y-axis) against relative ranks (absolute rank divided by the number of genes; x-axis). Thick solid lines represent normed coefficients; thin solid lines the values of all genes (see Figure 1 in the main text) in order to highlight effects on m2/ARE regulated genes relative to all genes analyzed.

A

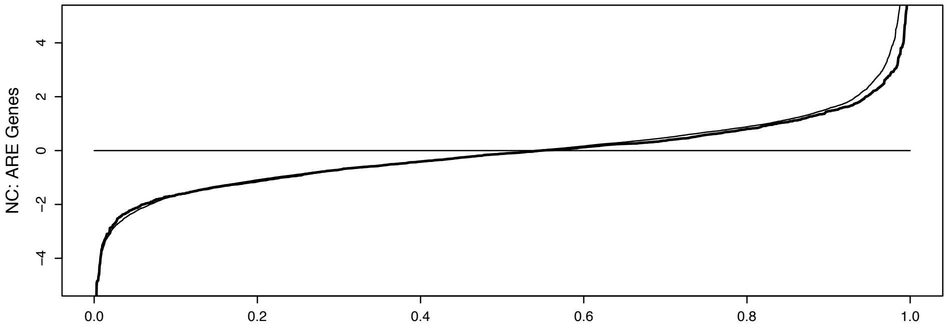

B

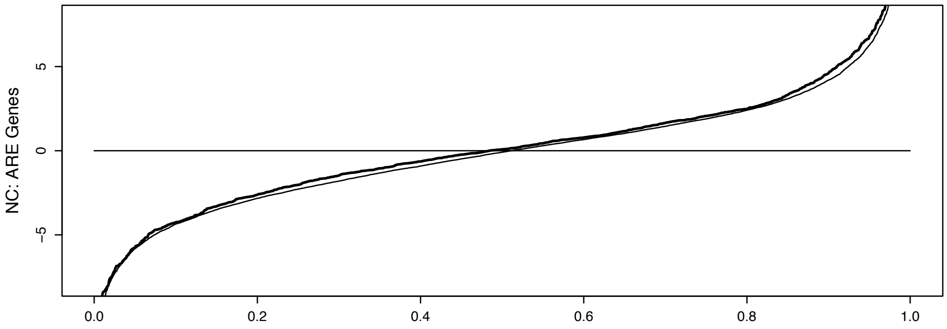

C

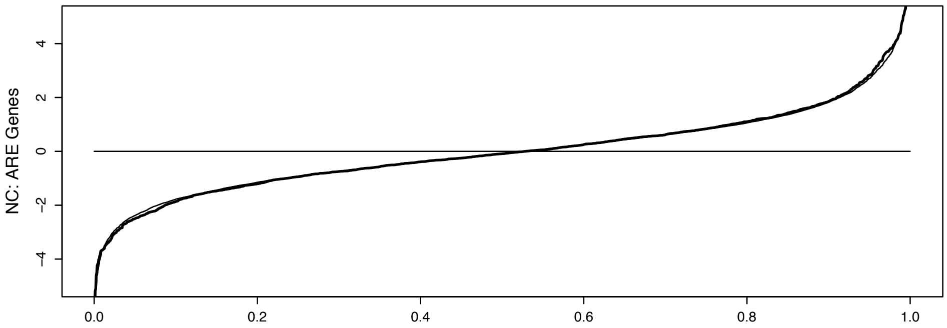

Supplement: Additional file 5 — Expression of genes with the m2/ARE 3'UTR regulatory sequence. This file contains a plot of the effects on expression levels of genes containing the m2/ARE 3'UTR regulatory sequence. [file 1471-2164-11-199-S5.PDF]
