# Supplementary material for: Transcriptome analysis reveals a major impact of JAK protein tyrosine kinase 2 (Tyk2) on the expression of interferon-responsive and metabolic genes
Source: BMC Genomics. 2010 Mar 25;11:199. doi: 10.1186/1471-2164-11-199 (PMC2864243; doi:10.1186/1471-2164-11-199)
Supplement: Additional file 6 — Analysis of 3'UTR miRNA cognate motifs. This file contains a list of genes with 3'UTR miRNA cognate sequences and a t-test analysis of their expression patterns. [file 1471-2164-11-199-S6.PDF]

Additional File 6

Analysis of 3’UTR miRNA cognate motifs

3’UTR motif	number of genes	ER	NC	p-value	miR [14]	miR (miRBase)
**(A)**						
o15.12	8	1.24	2.01	0.0005	miR-5	miR-18ab, miR-20ab
o15.22	10	1.17	1.47	0.0043	miR-94	miR-721
o28.5	14	0.86	-1.43	0.0030	miR-201	-
**(B)**						
o2.25	2	2.63	9.07	0.0011	miR-208(no.3)	-
o13.2	2	2.36	8.06	0.0035	miR-99a, miR-99b(no.3), miR-100	miR-99a,miR-100
o3.7	29	1.22	1.87	0.0033	miR-26	let-7d
**(C)**						
o2.25	2	1.99	4.08	0.0003	miR-208(no.3)	-
o13.2	2	1.75	3.34	0.0031	miR-99a, miR-99b(no.3), miR-100	miR-99a,miR-100
o46.1	17	1.20	1.07	0.0037	miR-221(no.1), miR-222(no.1)	

The table shows a *t*-test analysis for a total of 111 3’UTR miRNA cognate sequence motifs, identified as in Xie et al. [14]. Additionally, the sequence code as found by searching for the reverse complement in miRBase are shown. (A) Basal differences between genotypes, (B) LPS induction in WT, and (C) differences between genotypes in LPS induction (genotype by treatment interaction). ER, approximate mean expression ratios, NC, mean normed coefficients, and p-values from *t*-tests.
