# Supplementary material for: Transcriptome analysis reveals a major impact of JAK protein tyrosine kinase 2 (Tyk2) on the expression of interferon-responsive and metabolic genes
Source: BMC Genomics. 2010 Mar 25;11:199. doi: 10.1186/1471-2164-11-199 (PMC2864243; doi:10.1186/1471-2164-11-199)

Additional File 7

Comparison to microarray data of Thomas et al. [20]

Comparison between log2 fold changes in Thomas et al. [20] (x-axis) and normed (approximately standard normal) coefficients of the same genes from our study. (A) IFNβ-/- influenced genes versus Tyk2 genotype (WT minus Tyk2-/-), (B) log2 LPS induction in WT versus coefficients for LPS treatment in WT in our study (LPS minus control), and (C) log2 of the genotype by treatment interaction (difference in LPS induction between WT and IFNβ-/-) versus coefficients for interaction in our study (difference in LPS induction between WT and Tyk2-/-). The correlation in LPS induction in WT between Thomas et al. [20] and our study is 0.48, which is quite high, considering the different platforms.

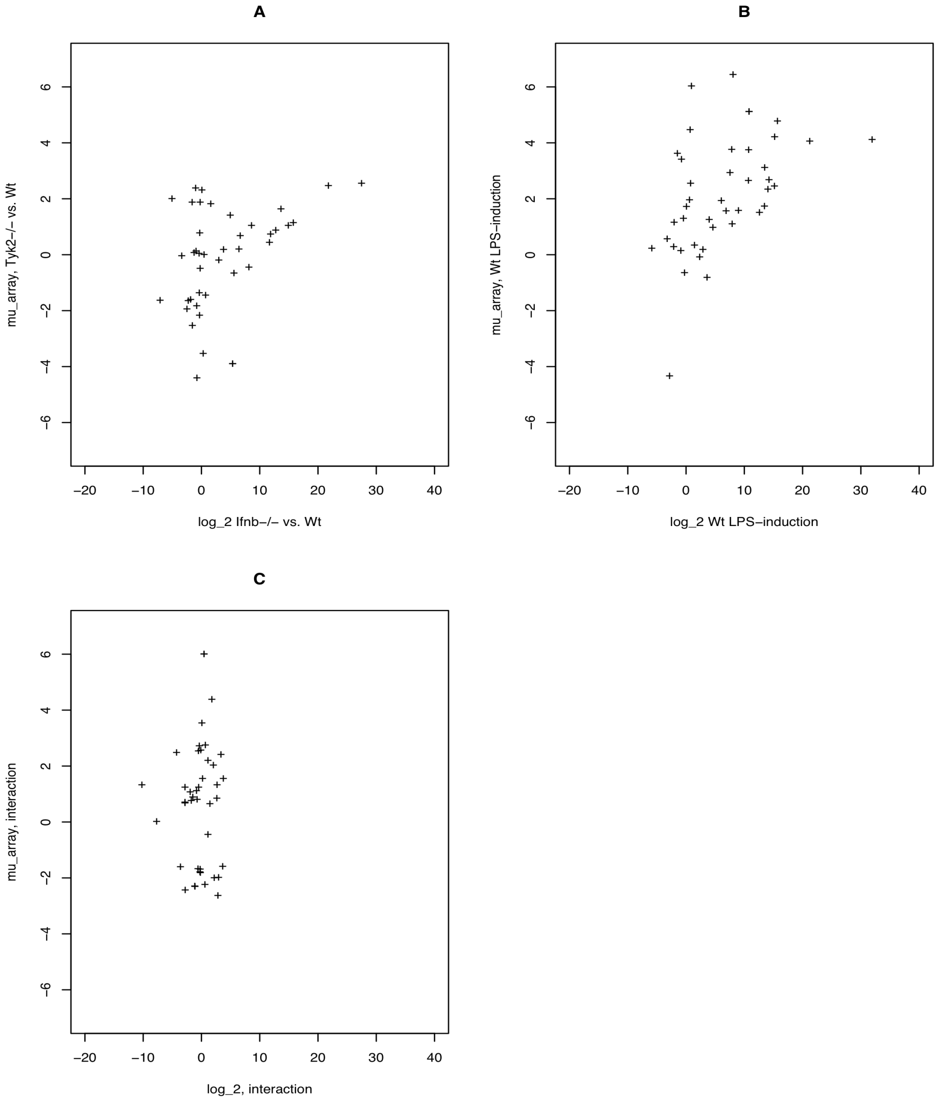

Supplement: Additional file 7 — Comparison to microarray data of Thomas et al. [20]. This file contains a graph depicting the relationship between the effects on expression of genes examined by Thomas et al. [20] and our study. [file 1471-2164-11-199-S7.PDF]
