# Supplementary material for: Transcriptome analysis reveals a major impact of JAK protein tyrosine kinase 2 (Tyk2) on the expression of interferon-responsive and metabolic genes
Source: BMC Genomics. 2010 Mar 25;11:199. doi: 10.1186/1471-2164-11-199 (PMC2864243; doi:10.1186/1471-2164-11-199)
Supplement: Additional file 8 — Comparison to microarray data of Fleetwood et al. [21]. This file contains a graph depicting the relationship between the effects on expression of genes examined by Fleetwood et al. [21] and our study. [file 1471-2164-11-199-S8.PDF]

Additional File 8

Comparison to microarray data of Fleetwood et al. [21]

Gene_symbol	NC_gt	NC_LPS	NC_int
Irf7	27.46	6.88	-10.23
Ccl5	3.73	16.55	1.63
Ly6e	13.58	2.74	-0.46
Stat2	8.58	10.69	-0.11
Stat1	13.65	7.91	-2.84
Slpi	-1.21	1.33	-1.75
Daxx	6.66	14.04	3.36
Gas7	0.40	5.74	-1.30
Mrc1	0.96	-3.33	-0.19
Ccrl2	2.65	15.25	1.97
Tlr7	-0.46	8.14	3.52
Trim21	5.56	10.74	2.03
H2-Q5	1.01	3.13	1.21
Cx3cl1	-0.95	0.09	-0.28
Arhgef10	1.27	1.16	0.21

List of genes downregulated in IFNAR1-/- genotypes in Fleetwood et al. [21] and corresponding normed (approximately standard normal) coefficients of the same genes in our study. Abbreviations: NC_gt, basal level difference of the normed coefficients between Wt and Tyk2-/-; NC_lps, LPS induction in WT; NC_int, genotype x treatment interaction in our experiment. Absolute values greater than two are significant at $p < 0.05$, and absolute values greater than three are significant at $p < 0.01$.
