# Supplementary material for: Transcriptome analysis reveals a major impact of JAK protein tyrosine kinase 2 (Tyk2) on the expression of interferon-responsive and metabolic genes
Source: BMC Genomics. 2010 Mar 25;11:199. doi: 10.1186/1471-2164-11-199 (PMC2864243; doi:10.1186/1471-2164-11-199)
Supplement: Additional file 9 — Validation of comparative approach to identify TFBS. This file contains a bioinformatic validation of the comparative approach to identify 5'UR TFBSs using publicly available microarray gene expression data. [file 1471-2164-11-199-S9.PDF]

Additional File 9

Validation of comparative approach to identify TFBS

To validate our comparative genomics approach for identifying TFBSs in the 5'UR, we used publicly available microarray data (http://symatlas.gnf.org/SymAtlas/) on samples from diverse mouse tissues. The expression profiles of all genes in the different tissues were clustered with the k-means algorithm. For further analysis, we selected two of these gene clusters: liver-specific genes, expressed preferentially in liver tissue, and muscle-specific genes, expressed preferentially in both muscle and heart. For each putative TFBS and each of the two tissues, a two by two contingency table was constructed: the TFBS is either present at least once within the LAGAN alignment of the first two kilobases of the 5'UR of mice and humans or is not present; and the gene is either present in the liver- or muscle-specific cluster, respectively, or not. These contingency tables were tested for enrichment of TFBSs with tissue-specific expression using a χ^2-test. In the liver specific gene-cluster, genes containing conserved TFBSs for HNF-1 (p-value: 10E-43) and HNF-3 (p-value: 10E-5) were significantly overrepresented; both p-values remained highly significant even after conservative Bonferroni correction for multiple testing. According to the Catalog of Tissue Specific Regulatory Motifs (TCat; http://rulai.cshl.edu/tcat/) these two transcription factors are also found to be highly liver-specific in humans, whereas only HNF-1 was found to be highly liver-specific in mice. In our analysis, human and mouse motives were searched jointly in the alignment, so that we cannot really distinguish between species. Determination of conserved TFBSs overrepresented in the muscle-specific gene cluster was more difficult, because this gene cluster showed also increased expression in heart tissue. The highest significantly overrepresented TFBSs in this cluster were MEF2 (p-value: 10E-40), AP4 (p-value: 10E-28), SRF (p-value: 10E-11), and MYOD (p-value: 10E-8). According to TCat, MEF2, AP4, and SRF are muscle-specific in humans and MYOD is heart-specific in humans. In conclusion, these results validate our method of detecting TFBSs.
